# Driving Environment Inference from POI of Navigation Map: Fuzzy Logic and Machine Learning Approaches

**DOI:** 10.3390/s23229156

**Published:** 2023-11-13

**Authors:** Yu Li, Martin Metzner, Volker Schwieger

**Affiliations:** 1 Institute of Engineering Geodesy, University of Stuttgart, Geschwister-Scholl-Str. 24D, 70174 Stuttgart, Germany; martin.metzner@iigs.uni-stuttgart.de (M.M.); volker.schwieger@iigs.uni-stuttgart.de (V.S.); 2 Daimler Truck AG, Fasanenweg 10, 70771 Leinfelden-Echterdingen, Germany

**Keywords:** driving environment inference, point of interest (POI), multilabel classification, fuzzy inference system, support vector machine, multilayer perceptron, navigation map

## Abstract

To adapt vehicle control and plan strategies in a predictive manner, it is usually desired to know the context of a driving environment. This paper aims at efficiently inferring the following five driving environments around vehicle’s vicinity: shopping zone, tourist zone, public station, motor service area, and security zone, whose existences are not necessarily mutually exclusive. To achieve that, we utilize the Point of Interest (POI) data from a navigation map as the semantic clue, and solve the inference task as a multilabel classification problem. Specifically, we first extract all relevant POI objects from a map, then transform these discrete POI objects into numerical POI features. Based on these POI features, we finally predict the occurrence of each driving environment via an inference engine. To calculate representative POI features, a statistical approach is introduced. To composite an inference engine, three inference systems are investigated: fuzzy inference system (FIS), support vector machine (SVM), and multilayer perceptron (MLP). In total, we implement 11 variants of inference engine following two inference strategies: independent and unified inference strategies, and conduct comprehensive evaluation on a manually collected dataset. The result shows that the proposed inference framework generalizes well on different inference systems, where the best overall $F_{1}$ score 0.8699 is achieved by the MLP-based inference engine following the unified inference strategy, along with the fastest inference time of 0.0002 millisecond per sample. Hence, the generalization ability and efficiency of the proposed inference framework are proved.

## 1. Introduction

**Figure 1:**
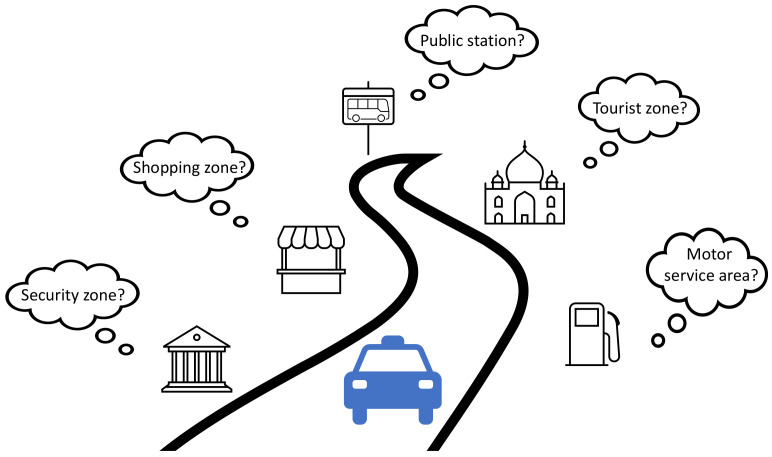
Using POI for driving environment inference.

Nowadays, environment perception plays an important role in automotive applications. One aspect of environment perception is to geometrically detect and track surrounding objects as precise as possible, to assist the driver to avoid potential collisions with other road obstacles. Such systems have been widely employed in Advanced Driver Assistance Systems (ADAS) applications such as Adaptive Cruise Control (ACC) [1] and Automatic Emergency Braking (AEB) [2]. Another aspect is to interpret the context of the driving environment as close to the reality as possible. Existing research has shown that knowing the context of driving environment can help to adapt the vehicle control and plan strategies in a more predictive manner. Example applications include intelligent vehicle power management [3,4,5,6], adaptive vehicle control [7,8,9,10,11,12], adaptive positioning [13,14,15], adaptive parametrization of perception algorithm [16,17], and fleet management [18]. In this paper, we focus on the inference of the following five driving environments around vehicle’s vicinity, i.e., a shopping zone, tourist zone, public station, motor service area, and security zone, which are mainly inspired by the use cases of the TransSec project [19]. As the semantic clue to address each driving environment, we utilize the Point of Interest (POI) data from a navigation map. Figure 1 graphically illustrates this idea.

To solve the driving environment inference problem, a variety of approaches have been developed within recent decades. Depending on the utilized data source, existing research can be divided into the following groups: vehicle-probe-data-based approaches, map-based approaches and vision-based approaches. To reduce fuel consumption and emission, the authors in [3,4,5,20] predicted road types (e.g., urban, rural, and highway roads) from onboard kinematic data such as vehicle speed and acceleration. Similarly, with the help of data mining techniques such as decision tree, Naive Bayes, and artificial neural network (ANN), other kinematic data such as gear position and wheel suspensions from CAN (Controller Area Network) bus can also be utilized to classify driving environments according to [8]. More recently, one noticeable method is proposed in [21], where the objective is to estimate the driving behavior and crash risk from onboard vehicle data such as speed, travel distance, and hand-on-wheel event. To achieve that, a variety of multiclass classifiers are investigated, such as Support Vector Machine (SVM), Random Forest, AdaBoost, and Multilayer Perceptron (MLP). Additionally, recent research has demonstrated the possibility to recognize different urban driving environments (e.g., open area, urban canyon, and tree shade) using various GNSS signal characteristics [13,14,15]. The basic idea behind these works is to utilize the statistical properties of historical GNSS signals as the feature, and then classify the driving environment using multiclass classifiers. Typically utilized classifiers include Support Vector Machine and other neural network approaches. Map-based applications are mostly focused on fuel economy; to achieve that, the road slope from map is utilized to identify the upcoming driving conditions [6,22,23]. Moreover, the POI data from map are also utilized by car insurance companies to predict the probability of car accident risk of their customers according to [24]. Vision-based approaches are applied in a wide range of applications, as they essentially take advantage of the advance in computer vision and pattern recognition over the recent years. Early vision-based approaches mainly utilize handcrafted image features for driving environment classification [7,9,25], while recent research has tended to solve this classification problem in an end-to-end fashion by leveraging modern neural networks [10,11,12,18,26]. As the common input to vision-based approaches, either the raw camera view or the so-called occupancy grid is utilized, where the occupancy grid can be calculated from LiDAR and/or radar measurement [11,12].

In general, the choice of data source depends on the environment types under investigation. For example, due to the legal speed limit differences between urban, suburban, and highway environments, vehicle-speed-related information provides delimiting hints to identify one driving environment from another [3,4,5,8,20]. In [6,22,23], the slope data from map are a key indicator for the upcoming road profiles such as uphill or downhill; therefore, it is considered as a proper choice. Camera view provides rich color and texture information about the environment, and hence, it is widely used in scene interpretations such as identifying urban versus rural roads, or minor versus major roads [7,9,10,11,12,26]. However, compared to existing research, the environment types in this work are unique in the following two senses. First, the five driving environments are semantically enriched by the functional properties of vehicle’s vicinity, i.e., each driving environment can be seen as a functional indicator of the nearby surroundings. Second, unlike the hard distinction between e.g., highway and urban environments, the existence of these five driving environments are not necessarily mutually exclusive, e.g., one road may belong to a shopping zone and a public station at the same time.

To solve the first problem, we use the POI data from a navigation map as the data source. Specifically, we use the concept "function" as the intermediate bridge between a POI object and a driving environment, and make the following assumptions: (1) one specific driving environment reflects a particular functional pattern of a location, which can be measured by the probabilistic existences of certain functions; (2) the occurrence of a specific POI object brings variable confidences to the existences of certain functions. With these assumptions, the intended inference can be seen as the process to numerically predict the existence of a specific driving environment from a given POI occurrence pattern. In fact, similar assumptions can also be found in References [27,28,29], where the intention is to automatically cluster and discover areas with similar functional properties. Despite that, these works also use map POI data as the main input, their focuses are mainly on large-scale geographical areas. As a result, the online processing capability is usually not required in these works, which is in contrary to the near-range and real-time demands in automotive applications. Regarding the data processing, due to the challenge in directly processing discrete POI objects, one usually needs to transform them into other representative POI features. For example, the author in [27] derived a POI feature vector to discover and annotate functional regions, where each term in this POI feature vector is calculated as the so-called POI frequency density measured by the number of a specific POI category over a unit area. A similar feature calculation method can also be found in [28], where the co-occurrence patterns of different POI categories are utilized to discover functional regions. Inspired by these works, in this paper, we propose a statistical feature calculation approach, which utilizes statistically calibrated POI occurrence patterns to quantitatively measure the confidence brought by the occurrence of certain POI objects to the existence of a specific driving environment.

As for the second problem, we propose to solve it as a multilabel classification task. In existing works, driving environment inference is generally solved as a classification problem [7,8,9,14,20,25]. Specifically, since the environment types are usually mutually exclusive in existing works, multiclass classifiers are often utilized as the ad hoc solutions. In contrast, in multilabel classification a sample is allowed to have more than one label, which is suitable for predicting the environment types that are not necessarily mutually exclusive. To solve the multilabel classification problem, it is common to transform the classification of multiple labels into a series of single-label classification subtasks, so that each subtask can be tackled by off-the-shelf classifiers [30]. Regarding the choice of classifier, it mainly depends on the structure of input data. For example, the Convolutional Neural Network (CNN)-based classifiers are frequently applied to handle image-like input [10,11,12,18,26]. Low-dimensional data such as the time series of vehicle probe data and the discrete map data are usually processed via other machine learning classifiers such as Support Vector Machine and Multilayer Perceptron (MLP) [3,13,14,20]. In our case, the classifier input is the calculated POI features, which is essentially a numeric vector with fixed dimension and size; thus, we consider the classic machine learning approaches as the classifier. Specifically, motivated by their success in classification tasks, we employ Support Vector Machine and Multilayer Perceptron as the classifier during implementation. Additionally, as another efficient tool that has been widely applied in spatial data analysis [31,32,33,34], the fuzzy inference system (FIS)-based classifier is also investigated in this work. It should be noted that, as the proposed inference framework is independent of the chosen classifier, one can in principle also employ other classifiers instead of these three.

In this paper, our objective is to develop an efficient inference framework that is capable of predicting the driving environments around vehicle’s vicinity. As the data source, we use solely the POI data from a navigation map. However, due to the difficulty in directly processing discrete POI objects, we propose a statistical approach to calculate representative POI features from raw POI objects. To accomplish the inference from POI features to a specific driving environment, we investigate the following three inference systems: fuzzy inference system, support vector machine, and multilayer perceptron. Particularly, we treat the driving environment inference task as a multilabel classification problem, and solve it through two inference strategies: the independent inference strategy and the unified inference strategy. To validate the proposed inference framework, we implement 11 inference engines and evaluate them on a manually collected dataset. In summary, with this work, we make the following contributions:

- A modular inference framework for the driving environment inference task with complete data processing workflows.
- A statistical feature calculation approach for the input transformation from discrete POI objects into semantically meaningful and numerically manageable POI features.
- The detailed composition of inference engines from three inference systems following two inference strategies.
- A comprehensive evaluation and comparison of 11 implemented inference engines on a manually collected dataset.

The remainder of this paper is organized as follows. Section 2 details the proposed inference framework, with particular focus on the proposed POI feature calculation method and the composition of inference engines from three investigated inference systems. Section 3 explains the implementation details and the experiment setups. Section 4 provides a comprehensive evaluation and comparison of 11 implemented inference engines. Finally, Section 5 concludes this paper and points out future directions.

## 2. Framework for Driving Environment Inference

### 2.1. Overview

By knowing the driving environment, the objective is to monitor and adapt the vehicle movement in a predictive manner. To achieve this goal, we conduct the inference based on a digital navigation map. Since our focus is on the vicinity of vehicle location, the problem can be translated to: given the vehicle GNSS position and a navigation map, how can we predict the driving environment(s) for the current vehicle location?

**Figure 2:**
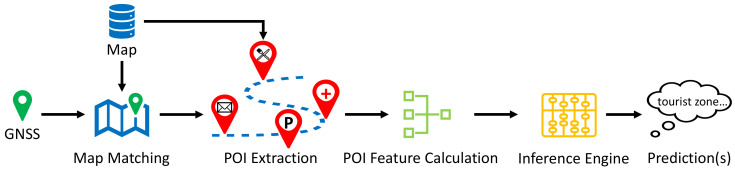
Overview of the proposed inference framework.

Figure 2 shows the overview of the proposed inference framework. This framework starts with map matching followed by the POI extraction process, where the purpose is to obtain the POI objects in vehicle’s vicinity. Then, based on the extracted POI objects, a POI feature calculation module is proposed to transform the discrete POI objects into numerical POI features that can be used for subsequent inference. Finally, an inference engine is built to predict the driving environment(s) at the given vehicle location. The remainder of this section is organized as follows. Section 2.2 provides an overview of the utilized navigation map, including a brief introduction of map matching and POI extraction within this map. Section 2.3 introduces a statistical approach for POI feature calculation. Finally, Section 2.4 details the compositions of inference engine using three inference systems: fuzzy inference system, support vector machine, and multilayer perceptron.

### 2.2. Navigation Map and Point of Interest Object

As the name suggests, a navigation map is a digital map that is built for navigating purposes. In automotive industries, the most popular navigation map format is the so-called Navigation Data Standard (NDS), which is developed by NDS e.V. [35,36,37]. NDS e.V. is a registered association and does not produce map data by itself; instead, it defines the map standard that is independent of navigation software. Digital maps complying with the NDS standard are called NDS map, which are usually produced by map suppliers such as HERE [38] and TOMTOM [39]. In addition to the basic geometry and topology of road network, a navigation map usually also contains other geo-referenced data. For example, a typical NDS map includes the following data blocks in its database: Routing block for road geometry and topology, POI block for geo-referenced places that can be selected as the navigation destination, and Name block for human references to certain locations and roads [35,40].

In navigation map, physical roads are typically represented by links and nodes, where a link stands for the road segment between two consecutive junctions and a node represents a road junction where two or more roads intersect [41]. Based on this link-node graph, one can match the vehicle position onto the map. This is usually achieved via the so-called map matching technique, which is essentially a process to find the best road candidate in the map given a series of vehicle positions (measured via, e.g., GNSS). The typical criteria for map matching include geometric point-to-line distance, topological connectivity, and the traversability between two roads [40,42,43].

Once the vehicle position is matched onto the map, the next step is to extract the nearby POI objects from the map database. Here, a practical question is the following: within which distance from the vehicle position should a POI object be considered as relevant for the inference? That is, if the distance is too large, the inference result may be diluted by the irrelevant POI objects that are far away. While a small distance may result in an insufficient number of extracted POI objects, i.e., too few POI objects to be representative. In either case, the inference result will not be able to reflect the actual driving environment in the vicinity. To solve this problem, one can either trim or extend the matched map link to a certain range according to the actual needs. For example, in our implementation, we set an upper length limit to trim single matched links that are too long, while we also selectively aggregate consecutive short links to form a long path if the matched link is too short. During this aggregation, we mainly utilize the most probable path (MPP) calculation logic to grow the ego path, where the commonly applied criteria include turn angle and the change of functional road class [44].

**Figure 3:**
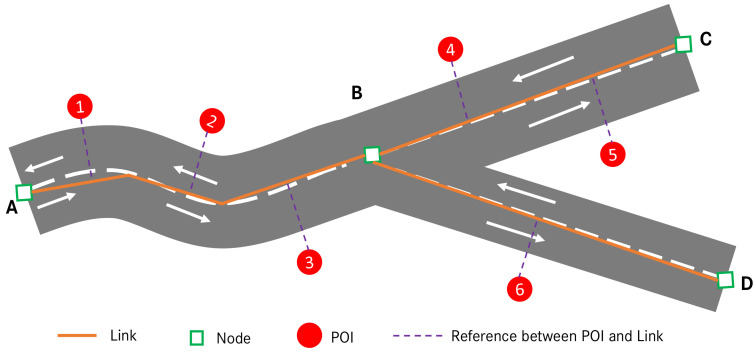
An example relation between link, node, and POI in NDS map. Note that each POI object is uniquely referred to a link from which it is accessible in reality.

Regarding the POI object in navigation map, it is usually stored as a single geolocation together with other supplementary attributes addressing its functional properties. For example, a restaurant is stored as a geolocation with the POI category “restaurant”, and possibly also with other information such as opening hours and contact details. Here, the POI category is important information to us, as it provides a semantic clue for predicting the functional property of the surroundings. Figure 3 depicts an example relation between link, node, and POI in NDS map.

As for the extraction of POI objects from map, it usually depends on the database structure of the utilized map. In NDS map, each POI object is uniquely referred to a certain link from which it is accessible in reality, see Figure 3. This is another important type of information in our application, as it allows to precisely query and extract all inherent POI objects for a given road link in map. For example, assume the vehicle is current located on link *AB* with the driving direction from *A* to *B*, and the MPP goes from link *AB* to link *BC* due to the smaller turn angle from *AB* to *BC*. Here, the vehicle’s vicinity is defined as the MPP that consists of link *AB* and link *BC*. Therefore, to extract all POI objects in the vicinity, we query from the map database all the POI objects that are accessible from link *AB* and link *BC*. As a result, we will obtain the following five POI objects *POI (1,2,3,4,5)*.

### 2.3. POI Feature Calculation: A Statistical Approach

In reality, the number of extracted POI objects may vary from one location to another. Besides, as we will see later in Section 2.4, all the investigated inference systems require continuous floating numbers as the input. Thus, directly processing the raw POI object with discrete POI categories is difficult, and we need to find an alternative. A general solution is to conduct the so-called feature engineering, which essentially creates new input variables (known as features) from the raw input source [45,46,47,48]. In our case, we consider the following two requirements on the new input variables: (1) the dimension and size of the new input variables should be numerically deterministic and (2) they should be representative and semantically meaningful for the intended inference. In this section, we first introduce the conceptual definition of POI features proposed in this paper, then we derive the mathematical calculation of these POI features.

To make the subsequent explanation easier, we define the following notations. Assume we have a training set $S=(p_{i},Y_{i}),1\leq i\leq n$, where *n* is the number of training samples. Each training sample $s_{i}\left(p_{i},Y_{i}\right)$ is a pair of input $p_{i}$ and target $Y_{i}$. $p_{i}$ is a vector of the extracted raw POI objects on sample $s_{i}$, and as discussed before, its size $|p_{i}|$ may vary over different samples. $Y_{i}$ is a binary vector of the ground truth labels: $Y_{i}=\left(y_{i}^{1},y_{i}^{2},\ldots ,y_{i}^{k}\right),Y_{i}\in {\left\{0,1\right\}}^{k},y_{i}^{j}\in \left\{0,1\right\},1\leq j\leq k$, where k=|L| is the number of unique labels in the investigated problem, and $L=\{\lambda_{1},\lambda_{2},\ldots ,\lambda_{k}\}$ is a constant label set which equals to {*shopping zone, tourist zone, public station, motor service area, security zone*} in our case. The term “label” is a terminology widely used in multilabel classification, and it is equivalent to “driving environment” in this paper. $y_{i}^{j}=1$ means the corresponding label $\lambda_{j}$ on sample $s_{i}$ is true, otherwise false. It should be noted that, in our case, a training sample may contain more than one true labels, e.g., a road may belong to both tourist zone and public station at the same time in reality. With these notations, feature engineering can be seen as a process to find a transformation *t* so that X=t(p), where the input p is an unbounded POI vector, and the output X is a deterministic POI feature vector: $X=\left(x_{1},x_{2},\ldots ,x_{m}\right),X\in R^{m}$, with *m* being a constant value.

#### 2.3.1. Conceptual Definition of POI Features

In principle, a representative and semantically meaningful POI feature should help to identify one specific characteristic of a driving environment during inference. To conceptually define such POI features, we start with analyzing the distribution of POI categories over a specific driving environment.

For a specific label $\lambda_{j},1\leq j\leq k$, the aforementioned training set *S* can be divided into the following two groups: positive training set $S_{j}^{+}=\left\{\left(p,Y\right)|y^{j}=1\right\}$ and negative training set $S_{j}^{-}=\left\{\left(p,Y\right)|y^{j}=0\right\}$, with $S=S_{j}^{+}\cup S_{j}^{-}$ and $\emptyset =S_{j}^{+}\cap S_{j}^{-}$. In each of these two groups, we can enumerate the unique POI categories, and correspondingly, this will result in the following two sets: a set of positive POI categories $C_{j}^{+}$ and a set of negative POI categories $C_{j}^{-}$. $C=C_{j}^{+}\cup C_{j}^{-}$ is a unique set of all available POI categories in the training set *S*. It should be noted that $C_{j}^{+}$ and $C_{j}^{-}$ are not necessarily mutually exclusive, i.e., $\emptyset \neq C_{j}^{+}\cap C_{j}^{-}$. For example, let us say we have two distinct samples $s_{1}\left(p_{1},Y_{1}\right)$ and $s_{2}\left(p_{2},Y_{2}\right)$, where $s_{1}$ is a “shopping zone only” sample (i.e., $Y_{1}=\left(1,0,0,0,0\right)$) and $s_{2}$ is a “public station only” sample (i.e., $Y_{2}=\left(0,0,1,0,0\right)$). $s_{1}$ can be seen as a negative sample of “public station”, and likewise, $s_{2}$ can be seen as a negative sample of “shopping zone”. Then, let us assume POI objects of the category “café shop” exist in both samples $p_{1}$ and $p_{2}$, which is feasible since in reality one may find a café shop both in a shopping zone and in a public station. Hence, we see that the POI category "café shop" exists in both the positive and the negative samples of the label “shopping zone”, and analogously, it also exists in both the positive and the negative samples of the label “public station”.

**Figure 4:**
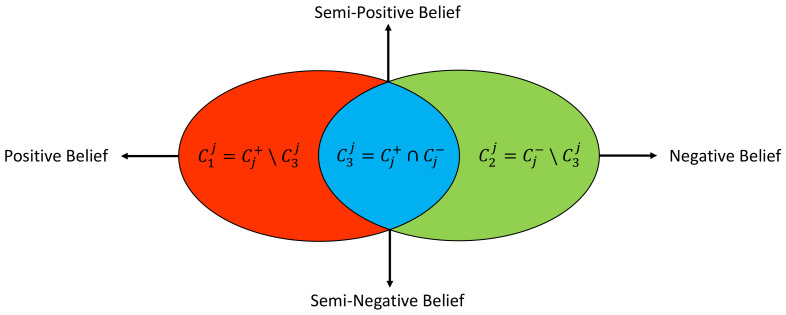
Four POI features derived from the distribution of POI categories over a specific driving environment $\lambda_{j}$.

Figure 4 graphically illustrates the distribution of POI categories over a specific driving environment $\lambda_{j}$. Apparently, for a specific driving environment $\lambda_{j}$, one POI category c∈C can only fall into one of the following three sets:

- Set 1: $C_{1}^{j}=\left\{c\right|c\in C_{j}^{+}$ *and* $c\notin C_{j}^{-}\}$, i.e., the POI object of category *c* exists only in the positive samples of label $\lambda_{j}$.
- Set 2: $C_{2}^{j}=\left\{c\right|c\notin C_{j}^{+}$ *and* $c\in C_{j}^{-}\}$, i.e., the POI object of category *c* exists only in the negative samples of label $\lambda_{j}$.
- Set 3: $C_{3}^{j}=\left\{c\right|c\in C_{j}^{+}$ *and* $c\in C_{j}^{-}\}$, i.e., the POI object of category *c* exists in both the positive and the negative samples of label $\lambda_{j}$.

Apparently, if an existing POI object belongs to $C_{1}^{j}$ or $C_{2}^{j}$, it can be utilized to uniquely identify a positive or negative $\lambda_{j}$ sample. While for a POI object of the group $C_{3}^{j}$, it can be used to identify both the positive and the negative samples of the same driving environment. In fact, even though one POI category may be intuitively linked to certain function, the interactions of different POI categories can reflect various functions [27,28]. Therefore, when a $C_{3}^{j}$ POI object is utilized to identify a positive/negative $\lambda_{j}$ sample, the underlying POI context should be considered. Based on these observations, we can define the following four POI features $x^{j}=\left(x_{1}^{j},x_{2}^{j},x_{3}^{j},x_{4}^{j}\right)$ for label $\lambda_{j}$:

- POI Feature 1 ($x_{1}^{j}$): *positive belief* from the POI objects that only exist in the positive samples of label $\lambda_{j}$. The higher this value, the more likely the corresponding driving environment exists.
- POI Feature 2 ($x_{2}^{j}$): *negative belief* from the POI objects that only exist in the negative samples of label $\lambda_{j}$. The higher this value, the less likely the corresponding driving environment exists.
- POI Feature 3 ($x_{3}^{j}$): *semi-positive belief* from the POI objects that exist in both the positive and the negative samples of label $\lambda_{j}$, which contributes to identifying positive $\lambda_{j}$ samples jointly with the *positive belief*.
- POI Feature 4 ($x_{4}^{j}$): *semi-negative belief* from the POI objects that exist in both the positive and the negative samples of label $\lambda_{j}$, which contributes to identifying negative $\lambda_{j}$ samples jointly with the *negative belief*.

The term *belief* can be seen as a degree of confidence, e.g., how confident it is to judge a sample of label $\lambda_{j}$ as positive/negative given the numerical value of the corresponding feature. To cover *k* labels in the inference task, we will have 4k POI features in total, as per the above definitions, i.e., the finally derived POI feature will be a vector of 4k dimensions (i.e., m=4k): $X=\left(x^{1},x^{2},\cdots ,x^{k}\right),X\in R^{4k},x^{j}\in R^{4},1\leq j\leq k$. In our case, since we have 5 labels (i.e., k=5), we will end up with a 20 dimensional POI feature vector (i.e., m=20).

#### 2.3.2. Mathematical Calculation of POI Features

Fundamentally, these four POI features are distinguished by their characteristic POI occurrence patterns. Once the characteristic POI occurrence pattern of a specific POI feature is known, the calculation of this POI feature can be seen as the numerical quantification of the similarity measure between a given POI occurrence pattern and a reference POI occurrence pattern. Now the question is, how can we mathematically define a POI occurrence pattern, and how should we model such similarity measure?

In our application, we have the following intuitions: (1) different POI categories can bring various degree of confidences when inferring the same driving environment, e.g., a shopping mall versus a grocery store when inferring the existence of shopping zone; (2) the number of occurrence of the same POI category can also change the degree of confidence during inference, e.g., ten grocery stores versus one grocery store when inferring a shopping zone. Based on these intuitions, we use POI occurrence probabilities to mathematically define a POI occurrence pattern, and a similar idea can also be found in [28]. Specifically, assume $N_{c}$ is the number of unique POI categories and w(c) represents the occurrence probability of the POI category c∈C, then the vector $w=\left(w\left(c_{1}\right),\ldots ,w\left(c_{N_{c}}\right)\right)$ uniquely defines a POI occurrence pattern. Since a POI occurrence pattern is now represented as a numerical vector, the similarity measure between two POI occurrence patterns can be addressed via the inner product of the corresponding vectors. Let $w_{l}^{j}=\left(w_{l}^{j}\left(c_{1}\right),\ldots ,w_{l}^{j}\left(c_{N_{c}}\right)\right)$ be the reference POI occurrence pattern of the POI feature $x_{l}^{j}$ on label $\lambda_{j}$, and let w be a given POI occurrence pattern, then the POI feature $x_{l}^{j}$ can be numerically determined as:

(1) $$x_{l}^{j}=w_{l}^{j}\cdot w,l\in \left\{1,2,3,4\right\}$$

For a given sample s(p,Y), if we approximate its POI occurrence pattern w by the POI occurrence counts, i.e., $w\approx \left(G_{s}\left(c_{1}\right),\ldots ,G_{s}\left(c_{N_{c}}\right)\right)$, where $G_{s}\left(c\right)$ is a counting function which simply calculates the number of occurrence of the POI category *c* in the sample *s*, then Equation (1) can be rewritten into:

(2) $$x_{l}^{j}=\sum_{c\in C}w_{l}^{j}\left(c\right)G_{s}\left(c\right),l\in \left\{1,2,3,4\right\}$$

From Equation (2), we see that each POI feature is numerically determined by the following two variables: a POI category dependent weighting factor and the occurrence of a POI category in a sample. This coincides with the aforementioned two intuitions. Now the remaining question is: how should we determine these weighting factors? That is, how should we numerically determine the reference POI occurrence pattern for each POI feature? Theoretically, one can handcraft a reference POI occurrence pattern using the expert knowledge derived from widely acceptable data sources, such as dictionaries, encyclopedias, and the design and planning standards of a local government [29]. Alternatively, one can also experimentally derive a reference POI occurrence pattern from a set of training samples [49]. However, the first method may face the following challenges in our application:

- Due to the large variety of POI categories existing in map (e.g., 89 in our case), it is a nontrivial task to manually quantify the contribution of each POI category to a specific driving environment.
- Given the geographic diversity in terms of urban planning and construction, the reference POI occurrence pattern designed for one geographic region may not be directly applicable to another region.

Therefore, we employ the second method by proposing a statistical approach. Particularly, for each POI feature, we calculate its weighting factors based on the POI occurrence probabilities over a set of training samples. The detailed calculations are given as follows:

- Weighting factors for feature 1 (*positive belief*):
  (3) $$w_{1}^{j}\left(c\right)=\left\{\begin{array}{cc} \frac{1}{|S_{j}^{+}|}\sum_{s\in S_{j}^{+}}P_{s}\left(c\right), & \mathrm{if}\;c\in C_{1}^{j}\\ 0, & \mathrm{if}\;c\in C\setminus C_{1}^{j} \end{array}\right.$$
  where $P_{s}\left(c\right)$ is the occurrence probability of one POI category *c* in a sample $s\left(p,Y\right),s\in S_{j}^{+}$, which is calculated according to:
  (4) $$P_{s}\left(c\right)=\frac{G_{s}\left(c\right)}{\sum_{c^{\prime }\in C_{1}^{j}}G_{s}\left(c^{\prime }\right)}$$
- Weighting factors for feature 2 (*negative belief*):
  (5) $$w_{2}^{j}\left(c\right)=\left\{\begin{array}{cc} \frac{1}{|S_{j}^{-}|}\sum_{s\in S_{j}^{-}}P_{s}\left(c\right), & \mathrm{if}\;c\in C_{2}^{j}\\ 0, & \mathrm{if}\;c\in C\setminus C_{2}^{j} \end{array}\right.$$
  where $P_{s}\left(c\right)$ is the occurrence probability of one POI category *c* in a sample $s\left(p,Y\right),s\in S_{j}^{-}$, which is calculated according to:
  (6) $$P_{s}\left(c\right)=\frac{G_{s}\left(c\right)}{\sum_{c^{\prime }\in C_{2}^{j}}G_{s}\left(c^{\prime }\right)}$$
- Weighting factors for feature 3 (*semi-positive belief*):
  (7) $$w_{3}^{j}\left(c\right)=\left\{\begin{array}{cc} \frac{1}{|S_{j}^{+}|}\sum_{s\in S_{j}^{+}}P_{s}\left(c\right), & \mathrm{if}\;c\in C_{3}^{j}\\ 0, & \mathrm{if}\;c\in C\setminus C_{3}^{j} \end{array}\right.$$
  where $P_{s}\left(c\right)$ is the occurrence probability of one POI category *c* in a sample $s\left(p,Y\right),s\in S_{j}^{+}$, which is calculated according to:
  (8) $$P_{s}\left(c\right)=\frac{G_{s}\left(c\right)}{\sum_{c^{\prime }\in C_{3}^{j}}G_{s}\left(c^{\prime }\right)}$$
- Weighting factors for feature 4 (*semi-negative belief*):
  (9) $$w_{4}^{j}\left(c\right)=\left\{\begin{array}{cc} \frac{1}{|S_{j}^{-}|}\sum_{s\in S_{j}^{-}}P_{s}\left(c\right), & \mathrm{if}\;c\in C_{3}^{j}\\ 0, & \mathrm{if}\;c\in C\setminus C_{3}^{j} \end{array}\right.$$
  where $P_{s}\left(c\right)$ is the occurrence probability of one POI category *c* in a sample $s\left(p,Y\right),s\in S_{j}^{-}$, which is calculated according to:
  (10) $$P_{s}\left(c\right)=\frac{G_{s}\left(c\right)}{\sum_{c^{\prime }\in C_{3}^{j}}G_{s}\left(c^{\prime }\right)}$$

Essentially, the weighting factor of a specific POI category is statistically calculated as the averaged occurrence probability over the corresponding training samples. For each POI feature, we see that only the relevant POI categories have nonzero weighting factors, the weighting factors of all other irrelevant POI categories are set to zero. This implies that the occurrence of these irrelevant POI categories will have no influence on the numerical value of the corresponding POI feature.

### 2.4. Inference Engine

As the last step in the proposed inference framework, an inference engine is utilized to predict all potentially existing driving environment(s). Fundamentally, an inference engine can be seen as one realized solution to the multilabel classification problem. In this subsection, we first explain the motivation of solving the intended inference task as a multilabel classification problem, including two proposed inference strategies and the corresponding optimizations. Then, we detail the composition of inference engines based on three inference systems: fuzzy inference system, support vector machine, and multilayer perceptron.

#### 2.4.1. Driving Environment Inference as a Multi-Label Classification Problem

From the discussion in Section 2.3, we know that the intended inference task has the following characteristics: (1) each given sample may contain more than one ground truth labels and (2) the inference of each label can be seen as a binary classification problem, i.e., does a given sample belong to a specific label or not? These characteristics coincide with the properties of multilabel classification task, which is basically a form of supervised learning where the classification algorithm is required to learn from a set of instances, and each instance can belong to multiple classes; thus, it is able to predict a set of class labels for a new instance [30].

Following the notations introduced in Section 2.3, the inference task can be defined as: given a POI feature vector $X,X\in R^{4k}$, how can we develop an inference engine $f_{\theta }:R^{4k}\to {\left\{0,1\right\}}^{k}$ which is conditioned on parameter set θ, so that the predicted label vector $\hat{Y}=f_{\theta }\left(X\right),\hat{Y}=\left({\hat{y}}^{1},{\hat{y}}^{2},\ldots ,{\hat{y}}^{k}\right)$ is “close” to the ground truth label vector Y up to certain qualification measures (e.g., accuracy, precision). The process to find the optimal parameters for this inference engine is generally known as training, which is equivalent to optimizing the following objective equation:

(11) $$\theta^{*}=\underset{\theta }{arg min}L_{S}\left(f_{\theta }\left(X\right),Y\right)$$

where $f_{\theta }$ is the inference engine under investigation, (X,Y)∈S is a single training sample in the given training dataset *S*, $L_{S}\left(\hat{Y},Y\right)$ is the overall loss on the whole training dataset *S*, and $\theta^{*}$ is the optimal parameter set that minimizes the overall loss.

**Figure 5:**
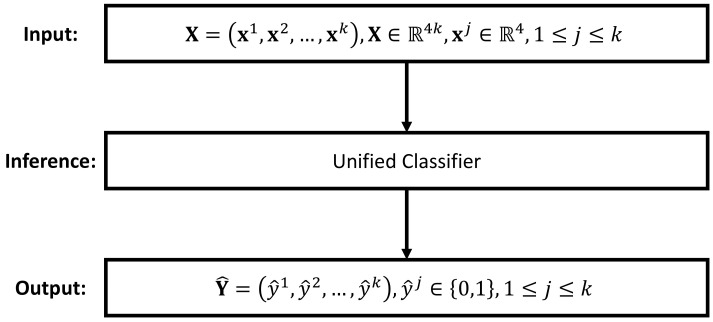
The proposed unified inference strategy.

**Figure 6:**
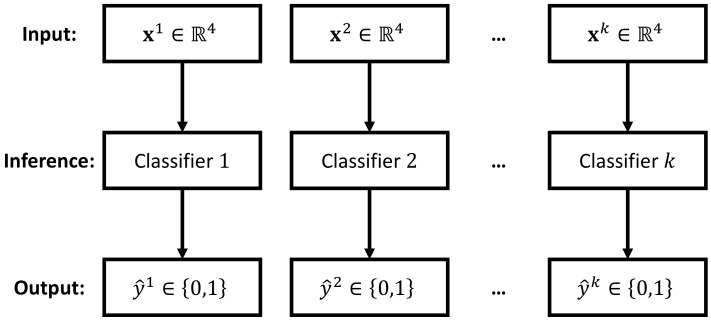
The proposed independent inference strategy.

To solve the multilabel classification problem, one common practice is to transform the classification of multiple labels into a series of single-label classification subtasks [30]. Depending on the utilized transformation method, difference inference strategies can be formed. In this paper, we propose the following two inference strategies: the independent inference strategy and the unified inference strategy. As depicted in Figure 5, the unified inference strategy aims at solving the inference task using a single classifier. This is achieved by training a *k*-output classifier, where each output represents the prediction for a specific label. In contrast, the idea of the independent inference strategy is to treat the inference of each label independently, so that a *k*-label multilabel classification problem can be solved by employing *k* independent classifiers. Figure 6 illustrates this idea. The advantage of the independent inference strategy is that any existing single label classifier can be directly applied for the inference task. However, in order to predict *k* labels, we need to implement *k* instances of such single classifier, which may theoretically increase the computational demand.

In the unified inference strategy, there is only one single classifier, and therefore, the optimization of the whole inference engine $f_{\theta }$ is identical to optimizing this single classifier, i.e., Equation (11). However, as for the independent inference strategy, there are *k* independent classifiers, as depicted in Figure 6. In this case, the optimization of an inference engine is equivalent to optimizing the following *k* independent equations:

(12) $$\theta_{j}^{*}=\underset{\theta_{j}}{arg min}L_{S}^{j}\left(f_{\theta_{j}}^{j}\left(x^{j}\right),y^{j}\right),1\leq j\leq k$$

where $f_{\theta_{j}}^{j}:R^{4}\to \left\{0,1\right\}$ is the classifier specified for label $\lambda_{j}$, $x^{j}$ is the label-specific POI feature vector calculated according to Equation (2), $y^{j}$ is the ground truth label for $\lambda_{j}$, $L_{S}^{j}\left({\hat{y}}^{j},y^{j}\right)$ is a label-specific loss function which calculates the overall loss caused by the classifier $f_{\theta_{j}}^{j}$ on the whole training dataset *S*, and $\theta_{j}^{*}$ is the optimal parameter set that minimizes this loss. In the independent inference strategy, a complete inference engine consists of *k* classifiers, i.e., $f_{\theta }=\left(f_{\theta_{1}}^{1},f_{\theta_{2}}^{2},\ldots ,f_{\theta_{k}}^{k}\right)$, which are conditioned on *k* sets of parameters, i.e., $\theta =(\theta_{1},\theta_{2},\ldots ,\theta_{k})$.

Fundamentally, the realization of a classifier is achieved via certain inference system. As next steps, we introduce three inference systems with particular focus on their integration and formulation into an inference engine by following the proposed inference strategies.

#### 2.4.2. Fuzzy-Inference-System-Based Inference Engine

Fuzzy inference system (FIS) is an inference system that is built upon fuzzy logic, and fuzzy logic is a logic system that aims at a formalization of approximate reasoning [50,51]. In contrast to the bivalent classical logic where only absolute true or false are permitted, fuzzy logic provides an efficient way of modeling partial truth or the degree of truth. This property makes it widely applicable in problems such as control, classification, and other decision-making applications [34,50,52,53,54]. A typical fuzzy inference process involves mainly three steps: fuzzification, inference, and defuzzification. Depending on the actual implementation of these steps, different inference mechanisms exist, such as the Mamdani inference system [55] and the Sugeno inference system [56]. As a common choice both in practice and in the literature [50,53], we take the Mamdani inference system as our investigation target and explain its principle.

Instead of working with the so-called crisp variables directly, fuzzy logic takes fuzzy set as the basic processing unit. A fuzzy set is a set with vague boundary between its members, and therefore, it can contain elements with only a partial degree of membership. Fuzzification is a process that transforms each input from a crisp value to a corresponding fuzzy input (i.e., a group of fuzzy sets), and this transformation is achieved via a series of predefined membership functions. A membership function (MF) is a numerical mapping from a point in the input space (also known as the universe of discourse) to a single value known as the grade of membership.

**Figure 7:**
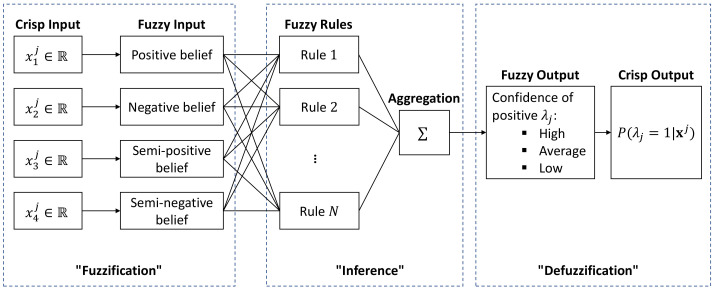
Fuzzy inference system for the inference of a single label $\lambda_{j},1\leq j\leq k$.

As an example, Figure 7 illustrates the inference process of a single label $\lambda_{j}$ in our application. In this case, the crisp inputs are four POI features calculated in Section 2.3: $x^{j}=\left(x_{1}^{j},x_{2}^{j},x_{3}^{j},x_{4}^{j}\right),x^{j}\in R^{4}$, and hence, the universe of discourse for each input is the real number set R. To comply with the definition of each POI feature, here, the fuzzy inputs are defined as the following four linguistic variables: “positive belief”, “negative belief”, “semi-positive belief”, and “semi-negative belief”. Analogously, the fuzzy output is defined as the linguistic variable “confidence of positive $\lambda_{j}$”. A linguistic variable is a variable whose values are words or sentences, where each word or sentence is generally known as a term which essentially represents a fuzzy set [53]. For each linguistic variable in our fuzzy inputs and fuzzy output, we define the following three terms: “high”, “average”, and “low”. Each term is numerically defined by a membership function on its corresponding crisp input/output. For example, the term “high” in the input linguistic variable “positive belief” is basically a fuzzy set defined by a pair of the crisp input $x_{1}^{j}$ and its membership value, which can be represented as:

(13) $$high=\{x_{1}^{j},\mu_{high}\left(x_{1}^{j}\right)|x_{1}^{j}\in R\}$$

where $\mu_{A}\left(x\right)$ is the membership function of a given crisp input *x* in the fuzzy set *A*. Each term requires one membership function, so we need in total 5×3=15 (5 linguistic variables times 3 terms in each linguistic variable) membership functions for the proposed FIS in Figure 7.

As the first step, fuzzification is the process to transform input from crisp values into fuzzy inputs, and this is achieved via a series of membership functions. Even though there exist research papers aimed at finding the proper membership functions for specific applications [57], it remains a flexible and mostly problem-oriented process, since the only requirement to a membership function is that its output should be a real number ranging between 0 and 1. Nevertheless, the commonly applied membership functions include: Triangular MF, Trapezoidal MF, Gaussian MF, combined Gaussian (cG) MF, and Bell-shaped MF [53]. Their mathematical expressions are defined in Equations (14)–(18), correspondingly. Here, $a,b,c,d,\sigma ,m,\sigma_{1},m_{1},\sigma_{2}$, and $m_{2}$ are the definitive parameters in the corresponding MF function; *x* is the input crisp value and μ(x) is the corresponding membership value:

(14) $$\mathrm{Triangular}\;\mathrm{MF}:\;\mu \left(x\right)=\max\left(\min\left(\frac{x-a}{b-a},\frac{c-x}{c-b}\right),0\right)$$

(15) $$\mathrm{Trapezoidal}\;\mathrm{MF}:\;\mu \left(x\right)=\max\left(\min\left(\frac{x-a}{b-a},1,\frac{d-x}{d-c}\right),0\right)$$

(16) $$\mathrm{Gaussian}\;\mathrm{MF}:\;\mu \left(x\right)=\exp\left[-\frac{1}{2}{\left(\frac{x-m}{\sigma }\right)}^{2}\right]$$

(17) $$\mathrm{cG}\;\mathrm{MF}:\;\mu \left(x\right)=\left\{\begin{array}{cc} \left\{\begin{array}{cc} \exp\left[-\frac{1}{2}{\left(\frac{x-m_{1}}{\sigma_{1}}\right)}^{2}\right], & \mathrm{if}\;x\leq m_{1}\\ 1, & \mathrm{if}\;m_{1}<x<m_{2}\\ \exp\left[-\frac{1}{2}{\left(\frac{x-m_{2}}{\sigma_{2}}\right)}^{2}\right], & \mathrm{if}\;m_{2}\leq x \end{array}\right., & \mathrm{if}\;m_{1}\leq m_{2}\\ \min\left(\exp\left[-\frac{1}{2}{\left(\frac{x-m_{1}}{\sigma_{1}}\right)}^{2}\right],\exp\left[-\frac{1}{2}{\left(\frac{x-m_{2}}{\sigma_{2}}\right)}^{2}\right]\right), & \mathrm{if}\;m_{1}>m_{2} \end{array}\right.$$

(18) $$\mathrm{Bell}-\mathrm{shaped}\;\mathrm{MF}:\;\mu \left(x\right)=\frac{1}{1+{\left|\frac{x-m}{\sigma }\right|}^{2a}}$$

As the second step, inference is a process where a series of fuzzy rules are evaluated and aggregated following certain fuzzy operations. A fuzzy rule is typically an If-Then conditional statement, which has the following form:

(19) If<antecedent>,Then<consequent>

where each antecedent is a premise which is built up on the terms of an input linguistic variable, and the consequent part is a conclusion acting on the terms of the output linguistic variable. One fuzzy rule may contain multiple antecedents that are connected with fuzzy operators. For example, one potential fuzzy rule for the proposed FIS in Figure 7 may look like: “If (positive belief is high) AND (negative belief is high) AND (semi-positive belief is high) AND (semi-negative is high), Then (confidence of positive $\lambda_{j}$ is high)”. In this case, the If-part consists of four antecedents that are joint via three intersection (AND) operators. In addition to intersection (AND), there exist other two fuzzy operators as well: union (OR) and complement (NOT). Assume $A\{x,\mu_{A}\left(x\right)\}$ and $B\{x,\mu_{B}\left(x\right)\}$ are two fuzzy sets, these three fuzzy operators are defined as follows:

(20) $$\mathrm{Intersection}\;(\mathrm{AND}):\;\left(A\cap B\right)\left\{x,\mu_{A\cap B}\left(x\right)\right\},\;\mathrm{where}\;\mu_{A\cap B}\left(x\right)=\min\left(\mu_{A}\left(x\right),\mu_{B}\left(x\right)\right)$$

(21) $$\mathrm{Union}\;(\mathrm{OR}):\;\left(A\cup B\right)\left\{x,\mu_{A\cup B}\left(x\right)\right\},\;\mathrm{where}\;\mu_{A\cup B}\left(x\right)=\max\left(\mu_{A}\left(x\right),\mu_{B}\left(x\right)\right)$$

(22) $$\mathrm{Complement}\;(\mathrm{NOT}):\;\bar{A}\left\{x,\mu_{\bar{A}}\left(x\right)\right\},\;\mathrm{where}\;\mu_{\bar{A}}\left(x\right)=1-\mu_{A}\left(x\right)$$

Theoretically, complex logic can be achieved by composing multiple simple fuzzy rules, which is generally known as fuzzy rule base. As long as a fuzzy rule base is constructed, the major task during inference is to evaluate all fuzzy rules. The evaluation of a fuzzy rule consists of two steps: (1) calculate the so-called degree of support for this fuzzy rule by aggregating all antecedents with the preselected fuzzy operators; (2) determine the consequent fuzzy set by truncating its membership function using the calculated degree of support. The second step is also known as the implication from antecedent to consequent [58]. Typically, each fuzzy rule only addresses a specific term of the output linguistic variable. Thus, we need to aggregate individual consequents into an overall consequent, so that it can be used to determine the final fuzzy output. For that, we apply the disjunctive operation “OR” as the aggregation method [50], which essentially conducts the union operation over all consequent fuzzy sets:

(23) $$C=(C_{1}\cup C_{2}\cup \ldots \cup C_{N})$$

where *C* is the output fuzzy set, $C_{j},1\leq j\leq N$ is the consequent fuzzy set from the fuzzy rule *j*, and *N* is the total number of fuzzy rules in the fuzzy rule base.

As the last step, defuzzification converts the output from a linguistic variable to a crisp variable that is more meaningful for the interested application. For example, the defuzzification process in Figure 7 converts the output from the linguistic variable “confidence of positive $\lambda_{j}$” to a numerical value, which can be interpreted as the probability that the given sample is positive in label $\lambda_{j}$: $P(\lambda_{j}=1|x^{j})$. There exists many defuzzification methods in the literature, but the most prevalent one is the Centroid method according to [50,53]. In the Centroid method, the crisp output is defined as the projection of the geometric center formed by the membership function of the output fuzzy set onto the crisp axis, which can be numerically calculated according to:

(24) $$z^{*}=\frac{\int \mu_{C}\left(z\right)\cdot z\;dz}{\int \mu_{C}\left(z\right)\;dz}$$

where *C* is the output fuzzy set calculated in Equation (23), $\mu_{C}\left(z\right)$ is the output membership function of the desired crisp variable *z* in the output fuzzy set *C*, and $z^{*}$ is the finally determined crisp output, which ranges between 0 and 1.

Since the crisp output from Equation (24) can be interpreted probabilistically, the proposed FIS can be used as a probabilistic classifier. To determine the predicted class ${\hat{y}}^{j}\in \left\{0,1\right\}$ for the given sample $x^{j}$, a threshold value to the crisp output $z^{*}$ is needed. For example, when a threshold value of 0.5 is applied, ${\hat{y}}^{j}$ can be calculated by:

(25) $${\hat{y}}^{j}=\left(z^{*}>0.5\right)$$

The depicted FIS in Figure 7 is essentially a single classifier, which can be directly plugged into Figure 6 to form an independent inference engine. With the above introduction, we can come up with the following observations on the fuzzy inference system:

- Membership function is an important component in fuzzy logic, as it bridges the gap between a crisp variable and the corresponding fuzzy set. In practice, the choice of proper membership function is treated as a hyperparameter, which needs to be fine-tuned in order to achieve the best inference performance.
- A properly designed fuzzy rule base is the key to success in fuzzy logic. However, the number of possible fuzzy rules grows exponentially with respect to the number of fuzzy inputs. Assume a FIS has $Q_{1}$ input and $Q_{2}$ output linguistic variables, where each input and output linguistic variable has $M_{1}$ and $M_{2}$ terms, correspondingly. Additionally, assume there is only one fuzzy operator type in the If-part. Then, the number of all possible fuzzy rules $N_{max}$ is equivalent to the permutation and combination of all input and output terms, which can be calculated as: $N_{max}=\left(M_{2}\cdot Q_{2}\right)\cdot \left({\left(M_{1}+1\right)}^{Q_{1}}-1\right)$. For example, the maximum number of possible fuzzy rules in the depicted FIS in Figure 7 is: $N_{max}=\left(3\cdot 1\right)\cdot \left({\left(3+1\right)}^{4}-1\right)=765$. If we adapt this FIS to the proposed unified inference strategy, i.e., by increasing both the crisp inputs and the fuzzy inputs from 4 to 20, and extending the fuzzy outputs and crisp outputs from 1 to 5, while still keeping 3 terms in each linguistic variable, then the maximum number of fuzzy rules will amount to: $N_{max}=\left(3\cdot 5\right)\cdot \left({\left(3+1\right)}^{20}-1\right)=16,492,674,416,625$. This makes the design of a proper rule base no longer practicable, even with the help of the existing software tools with automatic rule-learning capability like the MATLAB Fuzzy Logic Toolbox [59]. Such a data-dimension-related challenge is generally known as the curse of dimensionality [47,60].

#### 2.4.3. Support-Vector-Machine-Based Inference Engine

In the domain of classification, one of the most flexible and effective machine learning approaches is the support vector machine (SVM) [45,47,54]. Based on clear geometric intuition, the support vector machine has well-developed mathematical foundations in solving the two-class linear classification problem. Moreover, nonlinear classification can also be effectively solved by SVM with the help of the so-called kernel trick [60].

**Figure 8:**
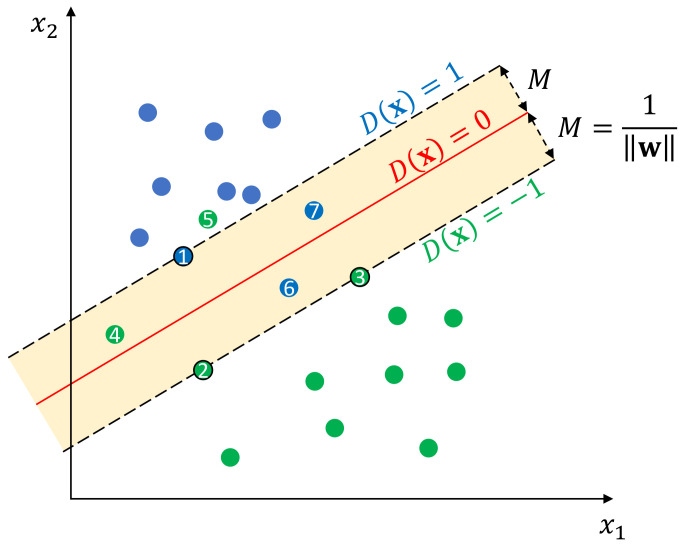
Support vector machine: margin and hyperplane.

Given a set of linearly separable training samples $\left\{\left(x_{1},y_{1}\right),\ldots ,\left(x_{n},y_{n}\right)\right\}$, where $x_{i}\in R^{d}$ is a *d*-dimensional input vector and $y_{i}\in \left\{-1,1\right\}$ is the corresponding class label, the target of support vector machine is to find a decision boundary in the input space $R^{d}$, so that samples of one class can be separated from the other. As shown in Figure 8, for linearly separable training samples, the decision boundary is actually a hyperplane in the input space $R^{d}$, which can be defined as:

(26) D(x)=w·x−b

where $w\in R^{d}$ and b∈R are the definitive parameters of this hyperplane, D(x)=0 represents the decision boundary itself, and D(x)=−1 and D(x)=1 represent the margin boundaries of class −1 and class 1, respectively. Margin is an important concept in SVM, which indicates the perpendicular distance between the decision boundary and the closest samples from each class. Margin *M* can be calculated as:

(27) $$M=\frac{1}{∥w∥}$$

In ideal case, the decision boundary shall separate all samples into the correct class, i.e., to the correct side of the decision boundary. That is, the following inequality should hold true for all training samples:

(28) $$y_{i}\left(w\cdot x_{i}-b\right)\geq 1,i=1,\ldots ,n$$

Hence, the goal of support vector machine is to find an optimal hyperplane in space $R^{d}$, which maximizes the margin in Equation (27) while satisfying the constraints in Equation (28). This is equivalent to solving the following optimization problem:

(29) $$\begin{array}{cc} \; & w^{*}=\underset{w}{arg min}\frac{1}{2}{∥w∥}^{2}\\ \; & \mathrm{subject}\;\mathrm{to}\;y_{i}\left(w\cdot x_{i}-b\right)\geq 1,i=1,\ldots ,n \end{array}$$

However, in practice, the class-conditional distributions may overlap, in which case exact separation of the training data can lead to poor generalization [60]. Therefore, a penalty term is usually added to Equation (29) to account for the loss introduced by the misclassified samples, e.g., samples 4,5,6, and 7 in Figure 8. To formulate this penalty term, a nonnegative slack variable $\xi_{i}\geq 0,i=1,\ldots ,n$ for each training sample is introduced, which is defined as the hinge loss: $\xi_{i}=\max\left(0,1-y_{i}\left(w\cdot x_{i}-b\right)\right)$. This slack variable will be 0 for samples lying on the correct side of the margin (including samples on the margin), while for other samples, this slack variable will grow linearly from 0 towards infinity depending on their geometric distances from the corresponding margin boundary. With this definition, the inequality in Equation (28) can be rewritten as:

(30) $$y_{i}\left(w\cdot x_{i}-b\right)\geq 1-\xi_{i},i=1,\ldots ,n$$

Accordingly, the optimization problem in Equation (29) is now updated to:

(31) $$\begin{array}{cc} \; & w^{*}=\underset{w}{arg min}\frac{1}{2}{∥w∥}^{2}+C\sum_{i=1}^{n}\xi_{i}\\ \; & \mathrm{subject}\;\mathrm{to}\;y_{i}\left(w\cdot x_{i}-b\right)\geq 1-\xi_{i}\;\mathrm{and}\;\xi_{i}\geq 0,i=1,\ldots ,n \end{array}$$

where C>0 is a regularization coefficient which controls the trade-off between the slack variable penalty and the margin loss during optimization. In contrast to the hard-margin optimization in Equation (29), the optimization task in Equation (31) is called soft-margin optimization, and the resulting hyperplane is called soft-margin hyperplane. It can be proved that, when *C* approaches infinity (i.e., C→∞), the optimizations in Equations (29) and (31) become identical.

In order to solve this constrained optimization problem, we can transform Equation (31) to the so-called dual space using the following Lagrangian function [60]:

(32) $$L\left(w,b,a,\mu \right)=\frac{1}{2}{∥w∥}^{2}+C\sum_{i=1}^{n}\xi_{i}-\sum_{i=1}^{n}a_{i}\left[y_{i}\left(w\cdot x_{i}-b\right)-1+\xi_{i}\right]-\sum_{i=1}^{n}\mu_{i}\xi_{i}$$

where $a=(a_{1},\ldots ,a_{n})$ and $\mu =(\mu_{1},\ldots ,\mu_{n})$; $a_{i}\geq 0$ and $\mu_{i}\geq 0$ are the Lagrange multipliers for each constraint in Equation (30) and for each slack variable $\xi_{i}$, respectively. Now, the problem is transformed to minimize the function L(w,b,a,μ) with respect to w and *b*, while maximizing it with respect to a and μ. To simplify the representation, we can substitute w, *b*, and μ with a by setting the derivatives of *L* with respect to w, *b*, and μ to 0. Consequently, Equation (32) is reformed into:

(33) $$\tilde{L}\left(a\right)=\sum_{i=1}^{n}a_{i}-\frac{1}{2}\sum_{i=1}^{n}\sum_{j=1}^{n}a_{i}a_{j}y_{i}y_{j}x_{i}\cdot x_{j}$$

Now the target is to find an optimal parameter vector a, which maximizes the quadratic equation in Equation (33) under the following derived constraints:

(34) $$\begin{array}{c} \sum_{i=1}^{n}a_{i}y_{i}=0\;\mathrm{and}\;0\leq a_{i}\leq C,i=1,\ldots ,n \end{array}$$

This is a convex optimization problem, which can be effectively solved by the quadratic programming algorithm with global convergence guarantee. However, the introduction to this algorithm is beyond the scope of this paper, and we refer the reader to [60,61,62] for further details. Once the parameter vector a is determined, the decision function in Equation (26) can be solved by:

(35) $$D\left(x\right)=\sum_{i=1}^{n}a_{i}y_{i}x_{i}\cdot x-b$$

Consequently, for a given sample with the input vector x, its predicted class $\hat{y}\in \left\{0,1\right\}$ is determined by checking the sign of the decision function D(x):

(36) $$\hat{y}=sign\left(D\left(x\right)\right)$$

In fact, the parameter vector a contains many zero entities, and only the nonzero entities have an effect on the final decision according to Equation (35). The training samples corresponding to these nonzero entities are known as support vectors, and hence, this technique is named support vector machine. For example, the samples 1,2, and 3 are the support vectors of the SVM depicted in Figure 8.

Up to now, all the discussions are based on the assumption that the given training samples are linearly separable in the input space. In cases where the samples cannot be separated by a linear classifier, SVM leverages the so-called kernel trick. The basic idea of the kernel trick is to convert the input vector from low dimension input space to a higher or infinite dimension feature space, in which the classification problem becomes tractable again by standard linear classifier. Commonly, such conversion is implicitly achieved using the so-called kernel function. A kernel function is a symmetric function which can be written as:

(37) $$k\left(x,x^{\prime }\right)=\phi \left(x\right)\cdot \phi \left(x^{\prime }\right)$$

where $x\in R^{d}$ and $x^{\prime }\in R^{d}$ are vectors in the input space and ϕ(x) is the nonlinear function that actually maps a vector from input space to feature space. The explicit representation of ϕ(x) is not necessary, as long as the output of the kernel function $k(x,x^{\prime })$ coincides with the inner product of this feature functions. One advantage of this kernel definition is that the theoretical development from Equation (27) to Equation (36) is still valid for the kernel-based nonlinear SVM classifier. For example, assume we have a linear kernel function $k\left(x,x^{\prime }\right)=x\cdot x^{\prime }$, i.e., ϕ(x)=x, Equation (26) can be rewritten as D(x)=k(w,x)−b=ϕ(w)·ϕ(x)−b, and thus, all the subsequent equation developments are still valid. Another advantage is that the computational effort of calculating the kernel function *k* is usually much less than naively constructing two ϕ(x) vectors and explicitly taking their inner product [47]. Commonly applied kernel functions include: linear kernel, polynomial kernel, radial basis function (RBF) or Gaussian kernel, and sigmoid kernel. Their definitions are given as follows:

(38) $$\mathrm{Linear}\;\mathrm{Kernel}:\;k\left(x,x^{\prime }\right)=x\cdot x^{\prime }$$

(39) $$\mathrm{Polynomial}\;\mathrm{Kernel}:\;k\left(x,x^{\prime }\right)={\left(\gamma x\cdot x^{\prime }+r\right)}^{d},\gamma >0$$

(40) $$\mathrm{RBF}/\mathrm{Gaussian}\;\mathrm{Kernel}:\;k\left(x,x^{\prime }\right)=\exp(-\gamma ∥x-x^{\prime }{∥}^{2}),\gamma >0$$

(41) $$\mathrm{Sigmoid}\;\mathrm{Kernel}:\;k\left(x,x^{\prime }\right)=\tanh\left(\gamma x\cdot x^{\prime }+r\right)$$

where γ,r, and *d* are the hyperparameters in the corresponding kernel function. Similar to the membership function in fuzzy inference system, the choice of proper kernel function is also a hyperparameter.

It should be noted that the introduced SVM is actually a decision machine, i.e., only the sign of the decision function is relevant for determining the final class. Therefore, the SVM-based classifier is a nonprobabilistic binary classifier. Since it is a binary classifier, a single SVM cannot model the joint optimization over multiple labels simultaneously. This is the reason why, currently, we only implement the SVM-based classifier into an independent inference engine.

#### 2.4.4. Multilayer-Perceptron-Based Inference Engine

Another popular machine learning approach is multilayer perceptron (MLP), which is essentially a feedforward neural network with fully connected nodes (also known as neurons) [47,53,60]. A multilayer perceptron consists of at least three layers of nodes, namely an input layer, a hidden layer, and an output layer. Except for the input nodes, each node in the hidden layer and the output layer represents a computational unit, which takes the outputs of the directly preceded layer as input and maps it nonlinearly into a scalar value that is usually known as the activation of this node. It has been proved that, even the simplest three-layer MLP is a universal approximator [63].

**Figure 9:**
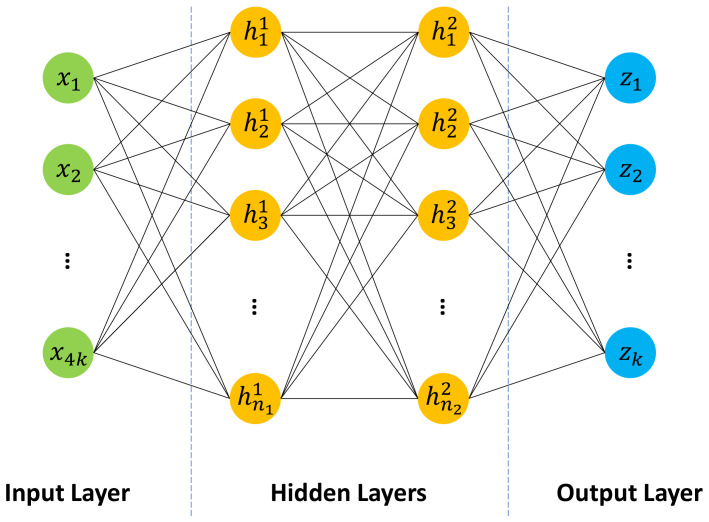
Example multilayer perceptron as a unified inference engine.

Figure 9 shows a four-layer multilayer perceptron, which is built as a unified inference engine for our inference task. In the input layer, each node stands for a single POI feature calculated in Section 2.3. Hence, the whole input layer can be numerically represented by the POI feature vector $X\in R^{4k}$, where *k* is the number of unique labels with the same meaning as in Section 2.3. The first hidden layer then takes this POI feature vector as input, and conducts the following operation:

(42) $$h_{1}=g\left(W_{1}X+b_{1}\right)$$

where $W_{1}$ is an $n_{1}$-by-4k weight matrix, $b_{1}$ is a $n_{1}$-dimensional bias vector, $h_{1}=\left(h_{1}^{1},\ldots ,h_{n_{1}}^{1}\right)$ is the output of the first hidden layer, an activation vector where $h_{i}^{1},1\leq i\leq n_{1}$ corresponds to the activation value of the *i*-th node in this layer, $n_{1}$ is the number of nodes in the first hidden layer, and g(·) is an element-wise activation function, which is chosen as the rectified linear unit (RuLU) function g(x)=max(0,x) in this paper. Apparently, the operation in a hidden layer is mathematically equivalent to an affine transformation followed by a nonlinearity transformation.

Likewise, the second hidden layer takes the activation vector from the first hidden layer as input and conducts a similar nonlinearity transformation:

(43) $$h_{2}=g\left(W_{2}h_{1}+b_{2}\right)$$

where $W_{2}\in M_{n_{2}\times n_{1}}R$ and $b_{2}\in R^{n_{2}}$ are the weight matrix and the bias vector of the second hidden layer, respectively, $n_{2}$ is the number of nodes in the second hidden layer, and $h_{2}=\left(h_{1}^{2},\ldots ,h_{n_{2}}^{2}\right)$ is the activation vector of the second hidden layer.

For each given sample, there are *k* unique labels to predict. Thus, we define *k* nodes in the output layer, where each node corresponds to a specific label. As a probabilistic classifier, we would expect each prediction to be a float number ranging between 0 and 1. Therefore, we employ the popular sigmoid function as the activation function in the output layer. It should be noted that the choice of the activation function is usually problem-oriented, e.g., linear activation function for regression, sigmoid activation function for binary classification, and softmax activation function for multiclass classification [47]. Consequently, the operation in the output layer can be written as:

(44) $$z=\sigma \left(W_{3}h_{2}+b_{3}\right)$$

where $W_{3}\in M_{k\times n_{2}}R$ and $b_{3}\in R^{k}$ are the weight matrix and the bias vector of the output layer, respectively, $z=\left(z_{1},\ldots ,z_{k}\right),z\in R^{k}$ is the prediction vector of the output layer, and σ(·) is the element-wise sigmoid function that is defined by:

(45) $$\sigma \left(x\right)=\frac{1}{1+\exp(-x)}$$

Each element in the prediction vector can be interpreted as the probability that its corresponding label is positive. Therefore, in order to determine the predicted binary label vector $\hat{Y}\in {\left\{0,1\right\}}^{k}$ introduced in Section 2.4.1, one can conduct the following element-wise “>” comparison to the prediction vector z. Note that 0.5 is an example threshold on each prediction:

(46) $$\hat{Y}=\left(z>0.5\right)$$

Equations (42)–(44) compose the so-called forward propagation of the proposed MLP, where all the weight matrices and bias vectors are the network parameters that need to be determined during training. Unlike support vector machine, the process of finding the optimal parameters for MLP is a nonconvex optimization problem [47], which cannot be solved by linear solvers. In practice, the training of neural network is usually achieved by using iterative and gradient-based optimizers, such as the stochastic gradient descent (SGD) algorithm [64,65]. The basic idea of stochastic gradient descent is to update the network parameters using the gradients of the loss with respect to the network parameters, and by doing such an update iteratively, the network parameters will finally converge to a certain optimal. During each iteration, the following updates are performed:

(47) $$W^{\left(\tau +1\right)}=W^{\left(\tau \right)}-\eta \nabla_{W}L\left(W,b\right)$$

(48) $$b^{\left(\tau +1\right)}=b^{\left(\tau \right)}-\eta \nabla_{b}L\left(W,b\right)$$

where τ denotes the iteration number, η is the learning rate, L(W,b) is the loss over a batch of training samples that is parametrized by the network weight W and bias b, and $\nabla_{W}L\left(W,b\right)$ and $\nabla_{b}L\left(W,b\right)$ represent the gradients of the loss *L* with respect to W and b, respectively. To calculate these gradients, the chain rule based back-propagation algorithm is usually applied [66]. It should be noted that, in addition to the standard SGD algorithm, there exists many other modern optimizers which not only consider the gradient itself, but also the momentum of each gradient over epochs. The advantage of utilizing momentum is that the resulting optimizer converges faster than the vanilla SGD optimizer. Example momentum-based optimizers include: Nesterov Momentum [67,68,69], AdaGrad [70], RMSProp [71], and Adam [72].

In terms of the loss function, we employ the weighted binary cross-entropy loss as our primary loss function, which is defined as follows:

(49) $$E\left(z,Y\right)=\sum_{i=1}^{k}\left[\alpha_{i}y_{i}\log\left(z_{i}\right)+\left(1-\alpha_{i}\right)\left(1-y_{i}\right)\log\left(1-z_{i}\right)\right]$$

where $Y\in {\left\{0,1\right\}}^{k}$ is the ground truth label vector as introduced in Section 2.3, $z\in R^{k}$ is the prediction vector calculated from Equation (44), $y_{i}\in \left\{0,1\right\}$ and $z_{i}\in R$ are individual elements in Y and z, respectively, $\alpha_{i}$ is a weighting factor to compensate the sample imbalance in each label, which ranges between 0 and 1, and *k* denotes the number of unique labels, as introduced in Section 2.3.

In addition to the primary loss, in practice, we often introduce a regularization loss on the network parameters to avoid the so-called overfitting of the network [73]. In this paper, we utilize the well-known L2 parameter norm penalty (also known as weight decay) as the regularization loss [47,48,60]. Assume θ is a vector representation of the network parameters W and b, then the final loss function can be defined as:

(50) $$\tilde{E}\left(z,Y\right)=E\left(z,Y\right)+\frac{\beta }{2}\theta^{⊤}\theta$$

where β is a regularization coefficient which acts as a weighting factor. It should be noted that the loss function in Equation (50) only calculates the loss of a single training sample, and one may need to sum up multiple such single losses to form a batch loss, e.g., the loss L(W,b) in Equations (47) and (48).

From the above introduction, we see that the depth (i.e., the number of layers) and width (i.e., the number of nodes in a specific layer) are two major considerations when designing a multilayer perceptron. Existing research has shown that deeper networks with fewer nodes have better generalization capability than shallow networks with wider layers, but deeper networks are often harder to optimize [47]. Therefore, to achieve a good balance, usually, intensive fine-tuning on network depth and width is performed. Furthermore, the flexible network design enables us to adapt the network architecture freely and quickly in practice. For example, we can easily modify the MLP in Figure 9 to the proposed independent inference strategy, e.g., by reducing the number of input nodes from 4k to 4, and keeping only a single node in the output layer.

## 3. Implementation and Experimental Setups

### 3.1. Implementation

**Figure 10:**
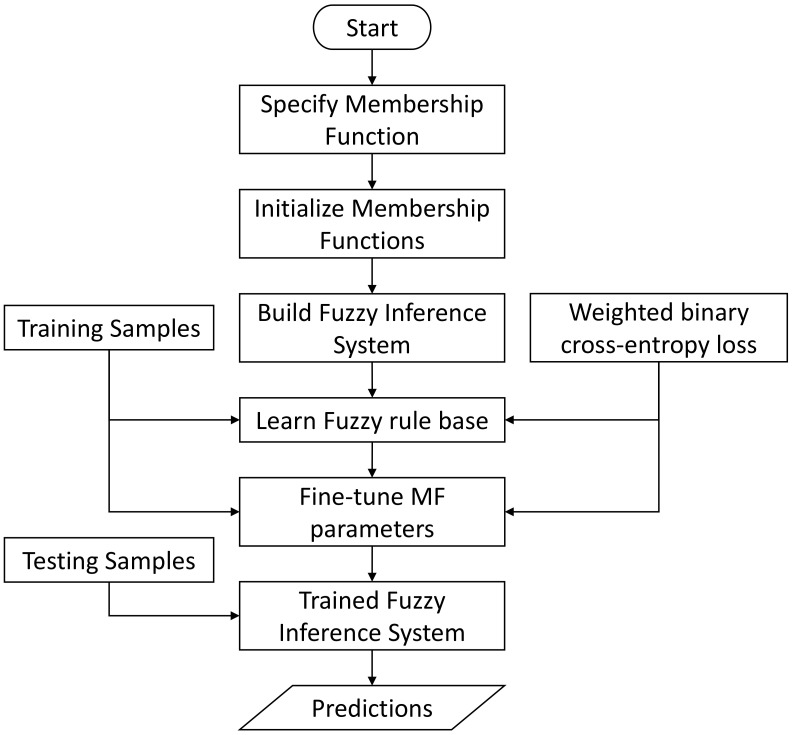
Implementation flowchart of a Fuzzy Inference System.

Due to the curse of dimensionality challenge introduced in Section 2.4.2, the implementation of FIS-based inference engine follows the independent inference strategy. In total, we implement five MF-specific inference engines, where each inference engine consists of five FIS-based classifiers to address the five driving environments correspondingly. The implementation of a Fuzzy Inference System is graphically illustrated in Figure 10. Once the type of membership function is specified, a default parameter set will be used to initialize all membership functions. Based on that, a Fuzzy Inference System is built. Then, the built Fuzzy Inference System will be trained using the given training samples. The training of a Fuzzy Inference System mainly includes learning a fuzzy rule base and fine-tuning all MF parameters. The trained Fuzzy Inference System can finally be used to make predictions for the testing samples during evaluation. To facilitate the training and evaluation, we use MATLAB as the programming language and leverage the existing MATLAB Fuzzy Logic Toolbox [59]. To learn a proper fuzzy rule base and to fine-tune the MF parameters in each classifier, MATLAB utilizes the genetic algorithm [53] as the optimizer. To guide the learning process, we employ the weighted binary cross-entropy loss in Equation (49) as the qualification measure. Furthermore, we set the threshold in Equation (25) to 0.5 for the final classification.

**Figure 11:**
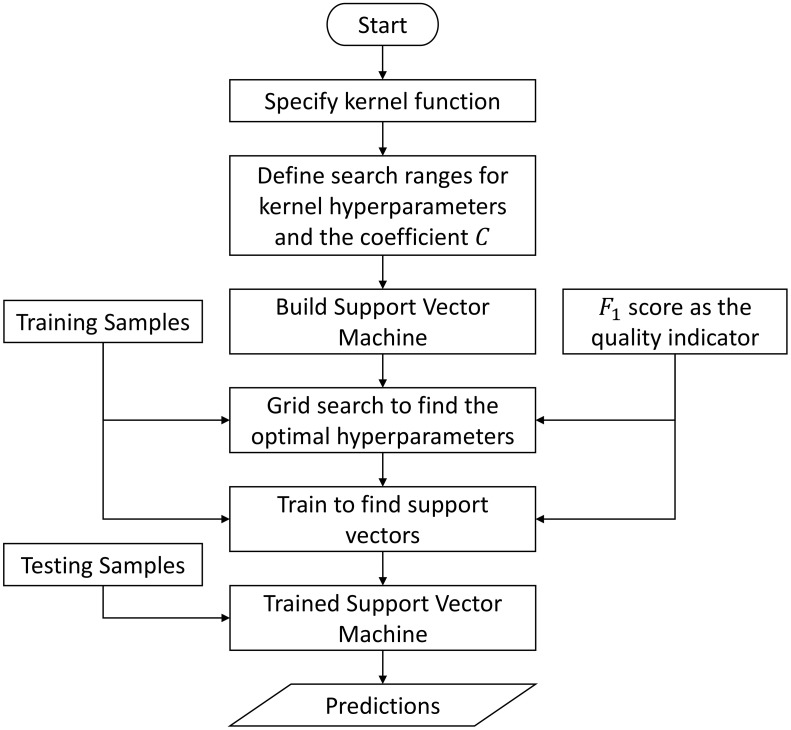
Implementation flowchart of a Support Vector Machine.

Given the discussions in Section 2.4.3, the implementation of an SVM-based inference engine also follows the independent inference strategy. In particular, we implement four kernel-specific inference engines, where each inference engine consists of five SVM-based classifiers to address the five driving environments correspondingly. All these SVM-based classifiers are implemented in Python, and the training and testing are achieved by leveraging the scikit-learn library (version 1.2.2) [74]. Figure 11 depicts the implementation flowchart of a Support Vector Machine. Since each SVM kernel function contains only nonlearnable hyperparameters, we conduct the so-called grid search to determine the optimal kernel hyperparameters and the regularization coefficient *C* [75]. The basic idea of grid search is that by evaluating the model performance over all possible hyperparameter combinations, we can finally find an optimal hyperparameter configuration that yields the best model performance. Therefore, for a chosen kernel function, the first step is to define the search ranges for the kernel hyperparameters and also for the regularization coefficient *C*. Once this is done, grid search will be conducted, and this is followed by the training process that finds support vectors from the training samples. As the quality indicator during these two processes, we utilize the $F_{1}$ score introduced in Equation (59). Finally, the trained Support Vector Machine can predict the existence of a specific driving environment for a given testing sample.

**Figure 12:**
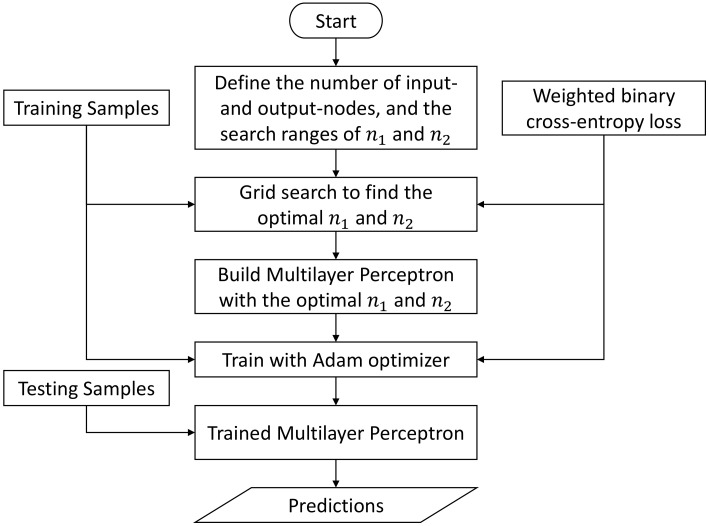
Implementation flowchart of a Multilayer Perceptron.

As for the multilayer perceptron, in addition to the unified inference engine depicted in Figure 9, we also implement an independent inference engine which comprises five independent MLP-based classifiers for five driving environments, correspondingly. As discussed in Section 2.4.4, the structural difference between a unified MLP and an independent MLP is on the number of input and output-nodes. Therefore, as shown in Figure 12, during the first implementation step, the number of input and output nodes should be defined. From Section 2.4.4, we also know that the depth and width of a network are the major architecture-relevant hyperparameters in MLP. To simplify our evaluation, in this paper, we fix the network’s depth as 4 (i.e., 1 input layer + 2 hidden layers + 1 output layer), and fine-tune only the widths ($n_{1}$ and $n_{2}$) of two hidden layers using grid search. Once the optimal $n_{1}$ and $n_{2}$ are found, a Multilayer Perceptron will be built. To train this Multilayer Perceptron, the gradient and momentum-based Adam algorithm is utilized as the optimizer, and the weighted binary cross-entropy loss as the loss function. Both the independent and the unified inference engines are implemented in Python, and we utilize the PyTorch library (version 2.0.0) [76] to facilitate the network design, training and evaluation. In terms of the threshold in Equation (46), we choose the same value 0.5 as in the Fuzzy Inference System.

In total, we implement 11 inference engines, which can be seen as 11 realization variants of the proposed inference framework. Despite the difference in programming languages, all the relevant training and testing tasks are performed on a laptop platform which runs an Intel Core i7-8750H CPU.

### 3.2. Evaluation Metrics

Since the driving environment inference task is solved as a multilabel classification problem, we employ standard multilabel classification metrics for the subsequent quantitative evaluation. Ref. [30] provides an overview of the commonly applied evaluation metrics in multilabel classification. In this paper, we consider the following five metrics: accuracy, precision, recall, $F_{1}$ score, and the false positive rate (FPR). Based on the notations introduced in Section 2.3 and Section 2.4.1, and assuming *Y* and $\hat{Y}$ are the set representations of the ground truth and the predicted label vectors Y and $\hat{Y}$, correspondingly, these five metrics can be defined as follows.

For a single sample, the accuracy is defined as the proportion of the correctly predicted labels over the total number (predicted and actual) of labels. The overall accuracy is then calculated as the average across all samples:

(51) $$\mathrm{Accuracy}:\;A=\frac{1}{n}\sum_{i=1}^{n}\frac{|Y_{i}\cap {\hat{Y}}_{i}|}{|Y_{i}\cup {\hat{Y}}_{i}|}$$

where $Y_{i}$ and ${\hat{Y}}_{i}$ are the ground truth and the predicted label sets of a single sample indexed by *i* and *n* is the total number of samples under evaluation.

For a single sample, the precision is defined as the proportion of the correctly predicted labels over the total number of predicted labels. The overall precision is then calculated as the average across all samples; note that $Y_{i}$, ${\hat{Y}}_{i}$, and *n* have the same meaning as in Equation (51):

(52) $$\mathrm{Precision}:\;P=\frac{1}{n}\sum_{i=1}^{n}\frac{|Y_{i}\cap {\hat{Y}}_{i}|}{|{\hat{Y}}_{i}|}$$

Similar to the precision, the recall for a single sample is defined as the proportion of the correctly predicted labels over the total number of actual labels, and the overall recall is then calculated as the average:

(53) $$\mathrm{Recall}:\;R=\frac{1}{n}\sum_{i=1}^{n}\frac{|Y_{i}\cap {\hat{Y}}_{i}|}{|Y_{i}|}$$

As a representative measure of both the precision and the recall, $F_{1}$ score is the harmonic mean of precision and recall, which is calculated according to:

(54) $$F_{1}\;\mathrm{score}:\;F_{1}=\frac{1}{n}\sum_{i=1}^{n}\frac{2|Y_{i}\cap {\hat{Y}}_{i}|}{|Y_{i}\left|+\right|{\hat{Y}}_{i}|}$$

Accuracy, precision, recall, and $F_{1}$ score are “goodness” measures, i.e., the higher their values, the better the performance of the investigated inference engine. In contrast, the false positive rate is a “weakness” measure, i.e., it reflects the probability to wrongly classify a negative label as positive for a given sample. The overall false positive rate across all samples can be calculated according to:

(55) $$\mathrm{False}\;\mathrm{Positive}\;\mathrm{Rate}:\;FPR=\frac{1}{n}\sum_{i=1}^{n}\frac{|{\bar{Y}}_{i}\cap {\hat{Y}}_{i}|}{|{\bar{Y}}_{i}|}$$

where ${\bar{Y}}_{i}$ is the complement set of $Y_{i}$, which represents all the negative ground truth labels on the sample indexed by *i*.

It should be noted that the metrics in Equations (51) to (55) are dedicated to reflect the overall classification performance across all *k* labels. In order to qualify the classifier capability on predicting a specific label, similar evaluation metrics are also needed. Assume $Y^{j},1\leq j\leq k$ is the ground truth label set on label $\lambda_{j}$ across *n* total number of samples, and correspondingly, ${\hat{Y}}^{j}$ is the predicted label set on label $\lambda_{j}$ across *n* total number of samples. Then, the individual evaluation metrics on single label $\lambda_{j}$ can be calculated analogously:

(56) $$\mathrm{Accuracy}\;\mathrm{on}\;\mathrm{label}\;\lambda_{j}:\;A^{j}=\frac{|Y^{j}\cap {\hat{Y}}^{j}|}{|Y^{j}\cup {\hat{Y}}^{j}|}$$

(57) $$\mathrm{Precision}\;\mathrm{on}\;\mathrm{label}\;\lambda_{j}:\;P^{j}=\frac{|Y^{j}\cap {\hat{Y}}^{j}|}{|{\hat{Y}}^{j}|}$$

(58) $$\mathrm{Recall}\;\mathrm{on}\;\mathrm{label}\;\lambda_{j}:\;R^{j}=\frac{|Y^{j}\cap {\hat{Y}}^{j}|}{|Y^{j}|}$$

(59) $$F_{1}\;\mathrm{score}\;\mathrm{on}\;\mathrm{label}\;\lambda_{j}:\;F_{1}^{j}=\frac{2|Y^{j}\cap {\hat{Y}}^{j}|}{|Y^{j}\left|+\right|{\hat{Y}}^{j}|}$$

(60) $$\mathrm{False}\;\mathrm{Positive}\;\mathrm{Rate}\;\mathrm{on}\;\mathrm{label}\;\lambda_{j}:\;FPR^{j}=\frac{|{\bar{Y}}^{j}\cap {\hat{Y}}^{j}|}{|{\bar{Y}}^{j}|}$$

In addition to these introduced metrics, we also report the model size (measured by number of parameters) and the inference time of each inference engine as an indicator of its computational efficiency.

### 3.3. Dataset

As the primary focus of the subsequent experiment is on the validation of the proposed POI feature calculation approach and the comparison of all implemented inference engines, therefore, we define each sample in the dataset as a pair of the POI feature vector $X,X\in R^{20}$ and the corresponding binary ground truth label vector $Y,Y\in {\left\{0,1\right\}}^{5}$. Specifically, in the first step, we manually collected and labelled 242 road samples in the area of Stuttgart, Germany. Then, we matched each road sample onto a NDS map by leveraging an existing map matching software. Once this matching is completed, we then extracted the corresponding POI objects for each road sample from the NDS map database using the approach introduced in Section 2.2. The NDS map used in this paper was compiled and released in 2017, which contains approximately 89 unique POI categories in total. Finally, based on the extracted POI objects, we calculated the POI feature vector X for each road sample following the proposed approach in Section 2.3.

As a result, our dataset contains 242 samples: $(X_{i},Y_{i}),1\leq i\leq 242$. During the following experiments, we split these 242 samples into 162 training samples and 80 testing samples. To improve the numerical stability during training and testing, we standardize each calculated POI feature in the training dataset to have 0-mean and the unit variance 1. Using the same standardization factors, we standardize the testing dataset as well.

## 4. Results and Discussion

### 4.1. Fuzzy-Logic-Based Driving Environment Inference

The training of each FIS-based classifier comprises two steps: learning a fuzzy rule base and tuning the parameters for all membership functions. In our experiment, we use the first 50 epochs to learn the fuzzy rule base. During this process, in order to keep the most principal fuzzy rules and to reduce the unnecessary computations on minor fuzzy rules, we limit the size of the target rule base to 30. After that, we fine-tune all MF parameters for 500 epochs. To avoid overfitting, we utilize the overall $F_{1}$ score as the quality indicator for early stopping. The evaluation results on testing dataset are summarized in Table 1, where the overall evaluation metrics correspond to the metrics introduced in Equations (51)–(55), and the individual evaluation metrics are calculated as the averages of Equations (56)–(60) across all five labels.

From Table 1, we can see that the inference engine specified by the combined Gaussian (cG) MF achieves the best performance across most of the “goodness” measures, both in terms of the overall and the individual evaluation metrics. Next to it is the Gaussian MF, which achieves 0.7850 and 0.8261 $F_{1}$ scores in the overall and the individual evaluation metrics, correspondingly. In fact, the Gaussian MF can be seen as a special case of the combined Gaussian MF, i.e., when $m_{1}=m_{2}$ and $\sigma_{1}=\sigma_{2}$, the combined Gaussian MF in Equation (17) will degenerate to the Gaussian MF in Equation (16). The Bell-shaped MF is, however, the worst-performing choice among the three nonlinear membership functions.

Compared with these three nonlinear membership functions, the two piecewise linear membership functions Triangular MF and Trapezoidal MF yield generally poor results on “goodness” measures, even though they have relatively better FPR measures both in the overall and the individual evaluation metrics. Moreover, the Trapezoidal MF based inference engine performs slightly better than that of the Triangular MF. In fact, Triangular MF can also be seen as a special case of the Trapezoidal MF, i.e., both membership functions are identical when b=c holds true in Equation (15).

In addition to the $F_{1}$ score, one may also focus on other quality indicators, such as precision, recall, and accuracy. Moreover, different applications may have special requirement on one set of evaluation metrics than the other. In these cases, Table 1 provides a reference for choosing the proper membership function for the driving environment inference task.

### 4.2. Support-Vector-Machine-Based Driving Environment Inference

**Figure 13:**
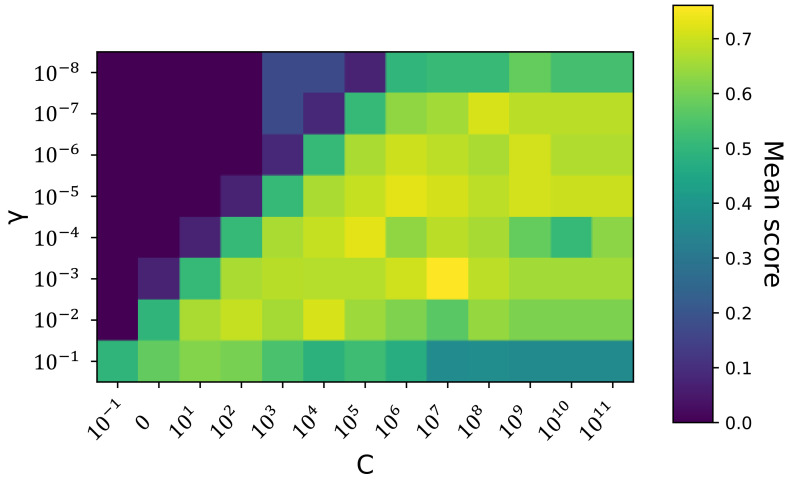
Grid search to find the optimal parameters *C* and γ for the RBF-Kernel-based SVM classifier.

The training of the SVM-based classifier is equivalent to solving the optimization problem in Equation (32), which is essentially a process to find all support vectors from the given training samples. In addition to that, another important aspect in SVM is to find the optimal model hyperparameters, which is usually achieved by grid search. Take the RBF Kernel based SVM classifier as an example; the model hyperparameters consist of the regularization coefficient *C* and the kernel-specific parameter γ. Following the suggestions in [75], we set the search ranges for *C* and γ as the exponentially growing sequences: $C=10^{-1},10^{0},\ldots ,10^{11}$, and $\gamma =10^{-8},10^{-7},\ldots ,10^{-1}$, correspondingly. We use $F_{1}$ as the scoring method, and calculate the mean $F_{1}$ score over three-fold cross-validations on the training dataset as the quality indicator at each grid. Figure 13 shows the grid search result of the SVM classifier trained for the label “shopping zone”, where the optimal values for *C* and γ are finally determined as $C=10^{7}$ and $\gamma =10^{-3}$.

This grid search has to be conducted for each single SVM classifier. Thus, in order to calibrate the five independent classifiers in each SVM-based inference engine, we need to run the grid search five times. Table 2 summarizes the performance of SVM-based inference engines on the testing dataset. It is notable that the overall performances of these four variants are generally close to each other. Nevertheless, the RBF Kernel yields relatively better results on the overall evaluation metrics, with the best achieved overall $F_{1}$ score of 0.8161. The Sigmoid-Kernel-based inference engine achieves the second best overall $F_{1}$ score of 0.8139, even though its individual $F_{1}$ score is lower than other three kernels. It is also worth to note that even the simple Linear Kernel based inference engine achieves comparatively good results among most evaluation metrics. When the $F_{1}$ score in the overall evaluation metrics is concerned, the worst performance of 0.8008 is achieved by the Polynomial-Kernel-based inference engine, but only with a difference of 0.0153 from the best overall $F_{1}$ score achieved by the RBF-Kernel-based inference engine.

### 4.3. Multilayer-Perceptron-Based Driving Environment Inference

**Figure 14:**
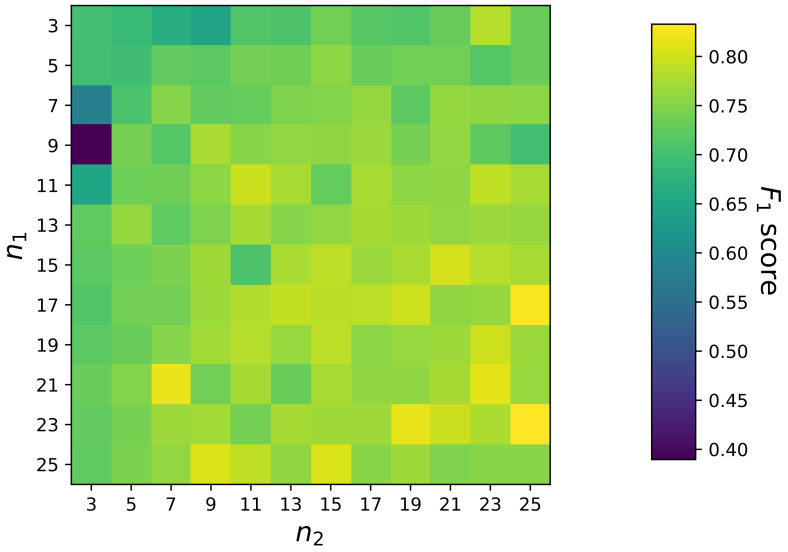
Grid search to find the optimal $n_{1}$ and $n_{2}$ for the unified inference engine in Figure 9.

During the training process, we start with a learning rate η of 0.0005, and reduce it by a factor of 5 once the learning stagnates. The target training epochs is set to 1500, during which the overall $F_{1}$ score is utilized for early stopping to avoid overfitting. As introduced in Section 3.1, to find an optimal MLP architecture, we conduct a grid search on the widths $n_{1}$ and $n_{2}$ of two hidden layers. During the grid search, we set the search ranges for $n_{1}$ and $n_{2}$ to be the same sequence: (3,5,…,23,25), and we use the overall $F_{1}$ score on training dataset as the quality measure. In our experiment, each search iteration is implemented as a standard training process with 1000 training epochs. Figure 14 shows the grid search result on the unified inference engine depicted in Figure 9, where the optimal values for $n_{1}$ and $n_{2}$ are finally determined as $n_{1}=23$ and $n_{2}=25$. Intuitively, the grid search result in Figure 14 shows slightly larger diversity along $n_{2}$ axis, e.g., the combination ($n_{1}=21$, $n_{2}=7$) yields almost the same performance as the combination ($n_{1}=23$, $n_{2}=25$). This implies that the optimal search of $n_{2}$ is comparatively harder than that of $n_{1}$. In practice, one can also increase the search ranges to have a broader overview of the $F_{1}$ landscape. However, increasing the search ranges also means the increase in computation demand.

Similar to the SVM-based inference engine, this grid search has to be done for each MLP classifier in order to find its optimal network architecture. Thus, we need to run this grid search five times for the independent inference engine and once for the unified inference engine. The performances of these two inference engines are summarized in Table 3. It is noticeable that the implemented unified inference engine surpasses the independent inference engine in all evaluation metrics. In fact, one implicit assumption of the proposed independent inference strategy is that the inferences of all target labels are mutually independent. However, in practice, this assumption might not always hold true according to the discussion in [30], and the result in Table 3 provides evidence for this. The proposed unified inference strategy yields better result, as it overcomes this assumption by implicitly modeling the label dependency within a single classifier.

### 4.4. Comparison of Inference Engines

From the results in Table 1, Table 2 and Table 3, we can draw the following conclusions:

- The MLP-based unified inference engine achieves the best overall performance among the 11 implemented inference engines, and the MLP-based independent inference engine generally yields better results than other independent inference engines. This shows, as a universal approximator, the superiority of the MLP-based inference system over the other two investigated inference systems.
- Both the FIS-based and the SVM-based inference engines show performance variances caused by the choice of different hyperparameters, e.g., the membership function in FIS and the kernel function in SVM. However, the performance variance in the FIS-based inference engines is comparatively larger than that in the SVM-based inference engines.
- Despite the aforementioned performance variances, with the calculated POI features, all three investigated inference systems are able to achieve a best individual $F_{1}$ score of more than 84%. This verifies the effectiveness of the proposed statistical POI feature calculation approach.
- Similarly, with the proposed inference framework, all the three investigated inference systems are able to achieve a best overall $F_{1}$ score of more than 81%. This proves the generalization ability of the proposed inference framework.

In addition to inference capability, Table 4 provides further comparisons regarding the inference efficiency. Here, the runtime is measured as the averaged inference time per sample, which is given in milliseconds. With the aforementioned software toolboxes, we see that the fastest inference engine (MLP-Unified) is about three orders of magnitude faster than the slowest inference engine (FIS-Triangular MF), even though it has about five times more trainable parameters. In general, the two MLP-based inference engines achieve the best runtime efficiency, and next to them are the SVM-based inference engines, where the linear kernel function tends to run faster than the nonlinear kernel functions. Conversely, all FIS-based inference engines show slower inference time than other two groups, which may be caused by the difference in the corresponding programming environments and software toolboxes. Given the fact that most of the current onboard vehicle positioning and map matching system has an update rate less than 50 Hz, and both the POI extraction and the POI feature calculation can be efficiently implemented on modern computer chips, we can conclude that even the slowest FIS-based inference engine (0.9310 ms/sample) is real-time capable. Thus, the proposed inference framework meets the real-time requirement.

In summary, MLP-based inference engine will be the primary choice for our inference task, when both the capability and the efficiency are desired. However, if the choice is a fuzzy inference system, then it is important to find the proper membership function in order to achieve the best inference result. In terms of the SVM-based inference engine, one may use the RBF Kernel function as a good starting point, while the simple Linear Kernel may achieve similar result but with less computational demand.

## 5. Conclusions

In this paper, we propose an inference framework to explore the feasibility of utilizing POI data for the driving environment inference task. The proposed inference framework mainly comprises four modules: map matching, POI extraction, POI feature calculation, and inference engine. The first two modules are designed to leverage the data structure of the utilized map, so that the purity of the extracted POI objects is guaranteed. Instead of working with discrete POI objects directly, we introduce a statistical approach to transform the input into semantically meaningful and numerically manageable POI features. Based on these POI features, an inference engine is built to solve the actual inference task. To realize that, we investigate the following three inference systems in this work: FIS, SVM, and MLP. Particularly, we detail the composition of inference engines from these three inference systems by following one of the two inference strategies: the independent inference strategy and the unified inference strategy. To examine the proposed inference framework, we implement 11 inference engines and evaluate them on a manually prepared dataset. The result shows that the proposed inference framework generalizes well over different inference systems, especially the configuration MLP-Unified achieves the best performance (overall $F_{1}$ score of 0.8699, with 0.0002 milliseconds of inference time per sample) among all implemented inference engines. Moreover, the effectiveness of the proposed POI feature calculation approach is also justified by the best-achieved individual evaluation metrics in each inference system. Last but not the least, the efficiency of the proposed inference framework is quantitatively demonstrated by the final efficiency comparison.

To correctly retrieve POI objects for the ego road being travelled, the proposed framework heavily relies on the map matching module. However, if map matching fails to match the vehicle location to the correct road link, then the inference result will no longer be reliable. As a potential solution, in the future we may try another POI extraction method, e.g., brute extraction of all POI objects within a certain range around a vehicle’s ego location. Besides, the proposed POI feature calculation method is essentially a data-driven approach. Similar to other data-driven approaches, a representative and unambiguous dataset is the key to success. However, obtaining such a dataset is usually challenging. To a certain degree, the introduced two inference strategies ensure the flexibility of the proposed framework, i.e., one can freely adapt existing inference systems to the proposed inference framework by following one of these two strategies. However, due to the introduced limitations adhere to FIS and SVM, currently, we only implement an MLP-based unified inference engine. As a future work, it is worth to validate the proposed inference framework using more inference systems, and also to investigate the extensibility of the introduced unified inference strategy to other inference systems. In addition to the current investigations on the three inference systems studied in this work, we will conduct more ablation experiments to further inspect the influence of other model-related hyperparameters on the final inference performance. For example, we can also apply the symmetric implicational method introduced in [77] to the fuzzy inference system. Finally, the POI source utilized in this paper is a commercial navigation map, although there exists other POI sources such as Google Maps and OpenStreetMap [78]. Therefore, another future topic is to further verify the proposed inference framework using other POI sources.

## Figures and Tables

**Table 1:** Performance of the FIS-based inference engines.

Inference Engine Specified by	Overall Evaluation Metrics					Individual Evaluation Metrics (Averaged)				
	**Accuracy**	**Precision**	**Recall**	**$F_{1}$ Score**	**FPR**	**Accuracy**	**Precision**	**Recall**	**$F_{1}$ Score**	**FPR**
Triangular MF	0.6438	0.7288	0.6928	0.6882	**0.0363**	0.6287	**0.8875**	0.6742	0.7610	0.0327
Trapezoidal MF	0.6716	0.7369	0.7190	0.7083	0.0394	0.6372	0.8857	0.7011	0.7717	**0.0319**
Gaussian MF	0.7059	0.8105	0.8301	0.7850	0.0713	0.7122	0.8633	0.8061	0.8261	0.0737
cG MF	**0.7565**	**0.8284**	**0.9085**	**0.8318**	0.0977	**0.7495**	0.8485	**0.8662**	**0.8497**	0.1054
Bell-shaped MF	0.6977	0.8121	0.7810	0.7645	0.0638	0.6595	0.8358	0.7529	0.7894	0.0566

**Table 2:** Performance of the SVM-based inference engines.

Inference Engine Specified by	Overall Evaluation Metrics					Individual Evaluation Metrics (Averaged)				
	**Accuracy**	**Precision**	**Recall**	**$F_{1}$ Score**	**FPR**	**Accuracy**	**Precision**	**Recall**	**$F_{1}$ Score**	**FPR**
Linear Kernel	0.7194	0.8095	0.9031	0.8121	0.0807	**0.7537**	0.8524	0.8753	**0.8504**	**0.0941**
Polynomial Kernel	0.7100	0.7933	0.8883	0.8008	0.0823	0.7310	0.8239	0.8687	0.8354	0.1159
RBF Kernel	**0.7279**	**0.8299**	0.8827	**0.8161**	**0.0788**	0.7402	**0.8593**	0.8452	0.8399	0.0949
Sigmoid Kernel	0.7206	0.8056	**0.9069**	0.8139	0.0899	0.7216	0.8000	**0.8786**	0.8269	0.1196

**Table 3:** Performance of the MLP-based inference engines.

Inference Engine	Overall Evaluation Metrics					Individual Evaluation Metrics (Averaged)				
	**Accuracy**	**Precision**	**Recall**	**$F_{1}$ Score**	**FPR**	**Accuracy**	**Precision**	**Recall**	**$F_{1}$ Score**	**FPR**
Independent	0.7647	0.8529	0.8922	0.8359	0.0706	0.7764	0.8846	0.8699	0.8680	0.0739
Unified	**0.8170**	**0.8824**	**0.9020**	**0.8699**	**0.0696**	**0.7986**	**0.8881**	**0.8895**	**0.8844**	**0.0674**

**Table 4:** Efficiency of the implemented inference engines.

Inference Engine	Number of Parameters		Runtime (ms/Sample)
	**Trainable**	**Hyperparameter**	
FIS (Triangular MF)	225	0	0.9310
FIS (Trapezoidal MF)	300	0	0.8897
FIS (Gaussian MF)	150	0	0.5387
FIS (cG MF)	300	0	0.5376
FIS (Bell-shaped MF)	225	0	0.5056
SVM (Linear Kernel)	0	0	0.0083
SVM (Polynomial Kernel)	0	20	0.0098
SVM (RBF Kernel)	0	10	0.0294
SVM (Sigmoid Kernel)	0	15	0.0120
MLP (Independent)	817	10	0.0007
MLP (Unified)	1213	2	0.0002

## Data Availability

The data presented in this study are available on request from the corresponding author.

## Abbreviations

The following abbreviations are used in this manuscript:

ACC	Adaptive Cruise Control
ADAS	Advanced Driver Assistance Systems
AEB	Automatic Emergency Braking
ANN	Artificial Neural Network
CAN	Controller Area Network
cG	combined Gaussian
CNN	Convolutional Neural Network
CPU	Central Processing Unit
FIS	Fuzzy Inference System
GNSS	Global Navigation Satellite System
MF	Membership Function
MLP	Multilayer Perceptron
MPP	Most Probable Path
NDS	Navigation Data Standard
POI	Point of Interest
RBF	Radial Basis Function
ReLU	Rectified Linear Unit
SGD	Stochastic Gradient Descent
SVM	Support Vector Machine
